# Trogocytosis and fratricide killing impede MSLN-directed CAR T cell functionality

**DOI:** 10.1080/2162402X.2022.2093426

**Published:** 2022-06-28

**Authors:** Esther Schoutrop, Stefanie Renken, Isabella Micallef Nilsson, Paula Hahn, Thomas Poiret, Rolf Kiessling, Stina L Wickström, Jonas Mattsson, Isabelle Magalhaes

**Affiliations:** a Department of Oncology-Pathology, Karolinska Institutet, Stockholm, Sweden; b Theme Cancer, Patient Area Head and Neck, Lung and Skin, Karolinska University Hospital, Stockholm, Sweden; c Department of Clinical Neuroscience, Karolinska Institutet, Stockholm, Sweden; d Gloria and Seymour Epstein Chair in Cell Therapy and Transplantation, Princess Margaret Cancer Centre and University of Toronto, Princess Margaret Cancer Centre, University Health Network, Toronto, Ontario, Canada; e Department of Clinical Immunology and Transfusion Medicine, Karolinska University Hospital, Stockholm, Sweden

**Keywords:** CAR T cells, mesothelin, trogocytosis, ovarian cancer, immune escape, fratricide killing

## Abstract

Successful translation of chimeric antigen receptor (CAR) T cell therapy for the treatment of solid tumors has proved to be troublesome, mainly due to the complex tumor microenvironment promoting T cell dysfunction and antigen heterogeneity. Mesothelin (MSLN) has emerged as an attractive target for CAR T cell therapy of several solid malignancies, including ovarian cancer. To improve clinical response rates with MSLN-CAR T cells, a better understanding of the mechanisms impacting CAR T cell functionality *in vitro* is crucial. Here, we demonstrated superior cytolytic capacity of CD28-costimulated MSLN-CAR T cells (M28z) relative to 4–1BB-costimulated MSLN-CAR T cells (MBBz). Furthermore, CD28-costimulated MSLN CAR T cells displayed enhanced cytolytic capacity against tumor spheroids with heterogeneous MSLN expression compared to MBBz CAR T cells. In this study, we identified CAR-mediated trogocytosis as a potential impeding factor for successful MSLN-CAR T cell therapy due to fratricide killing and contributing to tumor antigen heterogeneity. Moreover, we link antigen-dependent upregulation of LAG-3 with reduced CAR T cell functionality. Taken together, our study highlights the therapeutic potential and bottlenecks of MSLN-CAR T cells, providing a rationale for combinatorial treatment strategies.

## Introduction

Chimeric Antigen Receptors (CAR) are engineered transmembrane proteins that can re-direct lymphocyte specificity against tumor antigens. Combined with the activation of T cell effector functions, this allows for the eradication of tumor cells. CAR technology was pioneered over 30 years ago with the introduction of 1^st^ generation CAR T cells, containing an extracellular single chain variable fragment (scFv) and intracellular CD3 zeta-chain segment.*^1^* In 2nd-generation CARs, introduction of an intracellular co-stimulatory domain proved crucial for optimal CAR T cell survival, persistence, and robust anti-tumor responses.*^2,^^3^* Treatment with 2nd-generation CD19-directed CAR T cells has achieved successful therapeutic responses against refractory CD19+ malignancies, which ultimately resulted in FDA and EMA approval of four CD28- and 4–1BB containing CD19-CAR T cell products (tisagenlecleucel, axicabtagene ciloleucel, brexucabtagene autoleucel, and lisocabtagene maraleucel).^4–7^ However, CAR T cell effectiveness remains to be translated for solid tumors, largely due to hurdles such as the physical and immunosuppressive barriers of solid tumor microenvironment (TME) and limited availability of suitable target antigens.

Moving beyond the restricted expression of CD19 on the B cell lineage and nearly uniform expression on B cell malignancies, identifying ubiquitously expressed tumor-associated antigens in solid tumors with limited ‘on-target/off-tumor’ risk has proved complicated. Mesothelin (MSLN), among others, has emerged as a promising target for CAR T cell therapy due to its expression profile.^8^ MSLN, a GPI-anchored glycoprotein, is overexpressed in several solid tumors including pancreatic cancer, malignant mesothelioma, and ovarian cancer, while expression in healthy tissues is restricted to mesothelial cells. In ovarian cancer, MSLN has been found to be overexpressed in 55–97% of (high-grade) serous ovarian tumors.^9,10^ High-grade ovarian cancer is characterized by a complex tumor microenvironment, with buildup of malignant ascites and widespread (micro-) metastatic lesions across the peritoneal surfaces and omentum.^11,12^ Despite overexpression of MSLN, its expression is not homogenous within primary tumors and multifocal metastatic lesions of MSLN^+^ classified samples.^9,10^

In B cell malignancies, the loss or downregulation of surface antigen on tumor cells has been demonstrated to be a major mechanism of resistance against successful CD19-CAR T cell therapy.^13^ Recently, CAR T cell mediated trogocytosis has been reported to contribute to tumor antigen escape and CAR T cell dysfunction. Trogocytosis is an active process occurring at the immunological synapse, where membrane and membrane-associated molecules are transferred from antigen-presenting or target cells to effector cells.^14–16^ Trogocytosis-mediated loss of surface antigen poses yet another challenge to successful CAR T cell therapy of solid tumors expressing MSLN, as MSLN expression is naturally heterogeneous and trogocytosis may further lower expression. The induction of CAR-independent tumor cell lysis, referred to as ‘bystander killing’, could alleviate the lack of universal tumor antigen expression.^17,18^ To successfully translate CAR T cell therapy to solid tumors, elucidating the mechanisms affecting CAR T cell functionality *in vitro* is fundamental.

CD28 and 4–1BB are the most commonly used co-stimulatory domains and have been shown to differentially influence CAR T cell functionality, persistence, and cytolytic capacity *in vitro* and *in vivo*.^19–21^ We recently demonstrated enhanced persistence combined with reduced signs of exhaustion within the MSLN-CAR T cells carrying a 4–1BB co-stimulatory domain compared to CD28-containing MSLN-CARs in a preclinical *in vivo* orthotopic model of ovarian cancer.^22^ To overcome the difficulties posed by the complex high-grade serous ovarian cancer niche, it is crucial to investigate the mechanisms influencing CD28- and 4–1BB co-stimulated CAR T cell functionality.

In the present study, we compared effector functions and performed phenotypic analysis of MSLN-CAR T cells containing either a CD28 (M28z) or 4–1BB (MBBz) co-stimulatory domain in different *in vitro* models of ovarian cancer. Transduction with the M28z construct favored an effector T cell phenotype, while transduction with MBBz promoted outgrowth of central memory T cells. M28z and MBBz CAR T cells displayed antigen-dependent cytolytic activity and cytokine production against multiple MSLN^+^ target cells. M28z CAR T cells were superior in lysis of MSLN^+^ tumor spheroids compared to MBBz CAR T cells, regardless of the frequency of MSLN^+^ positive tumor cells (MSLN^low/mix/high^). Interestingly, both M28z and MBBz CAR T cells acquired the MSLN antigen through trogocytosis from ovarian cancer cells which, in turn, lost MSLN surface expression. Furthermore, we demonstrated MSLN-CAR T cell mediated fratricide killing of trogocytotic MSLN^+^ T cells.

## Material and methods

### CAR T cell production

Human peripheral blood mononuclear cells (PBMCs) were isolated from healthy donors (HDs) buffy coats (Karolinska University Hospital, Huddinge, Sweden). T cells were activated and subsequently transduced with γ-retroviral vectors encoding for two distinct MSLN-directed CAR constructs, M28z or MBBz, as reported previously.^23^ CAR T cells were cultured in AimV medium (Gibco, research grade) supplemented with 5% human serum (Karolinska University Hospital) and 300 IU/mL IL-2 (Novartis) unless stated otherwise. The CAR constructs encompass a human MSLN-specific scFv (m912,^24^) followed by either CD28-CD3ζ (M28z) or 4–1BB-CD3ζ (MBBz) signaling domains linked to truncated EGFR (EGFRt) (kindly provided by Prof. M. Sadelain, Memorial Sloan Kettering Cancer Center MSKCC, New York, USA). Production of γ-retroviral supernatant was performed according to previously published studies.^23,24^

### Human cancer cell lines

SKOV-3 human ovarian tumor cells (Adenocarcinoma, ATCC, HTB-77) were cultured in McCoy’s 5a medium (Sigma-Aldrich) supplemented with 10% fetal bovine serum (FBS, GE Healthcare) and 1% penicillin-streptomycin (Gibco). OVCAR-3 human ovarian tumor cells (Adenocarcinoma, ATCC, HTB-161) were maintained in RPMI-1640 (Gibco) supplemented with 20% FBS, 0,01 mg/mL insulin (Sigma-Aldrich) and 1% penicillin-streptomycin. K562 chronic myelogenous leukemia cells were included as control cells and they were kept in RPMI-1640 (Hyclone) supplemented with 10% FBS and 1% penicillin-streptomycin. Cells were retrovirally transduced with human MSLN variant 1 (SFG vector) and/or green fluorescent protein (GFP)/firefly luciferase fusion protein (SFG vector, both constructs gifted by Prof. M. Sadelain, MSKCC). Transduced SKOV-3, OVCAR-3, and K562 tumor cells were sorted by FACS (BD FACS aria, Becton Dickinson and SONY MA900 cell sorters, Sony Biotechnology Inc.) in order to isolate MSLN^high^GFP^+^ and MSLN^low^GFP^+^ polyclonal pools. The naturally MSLN devoid K562 cells were transduced with human CD19 and (GFP)/firefly luciferase fusion protein, followed by sorting to isolate CD19^+^GFP^+^ cells, and served as off-target control cells. Representative plots of MSLN% within parental and MSLN-transduced target cells can be found in Supplementary material 1.

### MACS beads sorting

M28z and MBBz CD4^+^ and CD8^+^ T cells were sorted through positive selection using CD4 microbeads, CD8 microbeads, and MS columns (all Miltenyi Biotec) according to the manufacturer’s instructions. Purity of positive selection products was assessed by FACS analysis (CytoFLEX, Beckman Coulter) and if required, the same sorting process was repeated. The sorted CD4^+^ and CD8^+^ CAR T cells were rested for 1–3 days at 37◦C and 5% CO_2_ prior to usage in CyQUANT lactate dehydrogenase (LDH) cytotoxicity (Thermo Fisher Scientific) assays.

### LDH cytotoxicity assays

Effector function of CD4^+^ sorted, CD8^+^ sorted, and mixed unsorted CD4^+^/CD8^+^ CAR T cells was determined by co-culture assays with MSLN^+^ transduced and MSLN_neg/low_ (untransduced) SKOV-3 and OVCAR-3 target cells. The frequency of transduced CAR T cells (EGFRt%) was determined prior to co-culture and adjusted to achieve a 2:1 and 1:5 CAR T cell to target cell ratio. Co-cultures were performed at 37◦C, 5% CO_2_ using AimV medium supplemented with 5% HS and without IL-2. Lysis of target cells was evaluated after 4 hours and 24 hours of co-culture by harvesting 50 µL of supernatant for CyQUANT LDH cytotoxicity assays according to manufacturer’s instructions. In addition, following four and 24 hours of incubation, cells from the 2:1 ratio were harvested for flow cytometry to assess phenotypic exhaustion and trogocytosis (Table 1). Approximately 50% of the cells harvested after 24 hours were used for flow-cytometry and 50% were transferred to a 48-well plate and rested in AimV supplemented with 5% human serum and 300 IU/mL IL-2 for 6 days, after which a new co-culture was started as described above at a 2:1 ratio for 24 hours.

**Table 1.** Flow cytometry stainings per panel.

Panel	Specificity	Conjugation	Vendor
Memory	EGFR (Cetuximab biosimilar)	Biotin	R&D systems, FAB9577B
	Streptavidin	PE	Biolegend®, 405204
	CD3	APC	BD Pharmingen™, 555335
	CD4	FITC	BD Pharmingen™, 555346
	CD8	APC-Cy7	BD Pharmingen™, 557834
	CD197 (CCR7)	PE-CF594	BD Pharmingen™, 562381
	CD45RA	PE-Cy7	BD Pharmingen™, 560675
	DNA	7AAD	BD Pharmingen™, 51–68981E
Exhaustion	Human FAB	PE	R&D systems, FAB9577B
	CD3	ECD	Beckman Coulter, A07748
	CD4	AF700	BD Pharmingen™, 557922
	CD8	APC-Cy7	BD Pharmingen™, 557834
	CD279 (PD-1)	FITC	BD Pharmingen™, 557860
	CD223 (LAG-3)	BV650	Biolegend®, 369316
	CD366 (TIM-3)	BV785	Biolegend®, 345032
	LIVE/DEAD™ Fixable Aqua		Invitrogen, L34957
Trogocytosis	Human FAB	RPE	Jackson Immunoresearch, 109–116- 097
	CD3	ECD	Beckman Coulter, A07748
	CD4	AF700	BD Pharmingen™, 557922
	CD8	APC-Cy7	BD Pharmingen™, 557834
	MSLN	APC	R&D systems, FAB32652A
	LIVE/DEAD™ Fixable Aqua	405 nm	Invitrogen, L34957
Functional trogocytosis	EGFR (Cetuximab biosimilar)	Biotin	R&D systems, FAB9577B
	Streptavidin	PE	R&D systems, FAB9577B
	CD3	PE-Cy7	BD Pharmingen™, 557851
	CD4	AF700	BD Pharmingen™, 557922
	CD8	APC-Cy7	BD Pharmingen™, 557834
	MSLN	APC	R&D systems, FAB32652A
	CellTrace™	Violet	Invitrogen^TM^, C34571
	DNA	7AAD	BD Pharmingen™, 51–68981E
Fratricide A & Bystander killing	EGFR (Cetuximab biosimilar)	Biotin	R&D systems, FAB9577B
	Streptavidin	PE	Biolegend®, 405204
	CD3	PE-Cy7	BD Pharmingen™, 557851
	CD4	AF700	BD Pharmingen™, 557922
	CD8	APC-Cy7	BD Pharmingen™, 557834
	MSLN	APC	R&D systems, FAB32652A
	CellTrace™	Violet	Invitrogen^TM^, C34571
	DNA	7AAD	BD Pharmingen™, 51–68981E
Fratricide B	EGFR (Cetuximab biosimilar)	Biotin	R&D systems, FAB9577B
	Streptavidin	PE	Biolegend®, 405204
	CD3	PE-Cy7	BD Pharmingen™, 557851
	CD4	AF700	BD Pharmingen™, 557922
	CD8	APC-Cy7	BD Pharmingen™, 557834
	MSLN	APC	R&D systems, FAB32652A
	CD279 (PD-1)	FITC	BD Pharmingen™, 557860
	CD223 (LAG-3)	BV650	Biolegend®, 369316
	CD366 (TIM-3)	BV785	Biolegend®, 345032
	CellTrace™	Violet	Invitrogen^TM^, C34571
	DNA	7AAD	BD Pharmingen™, 51–68981E
Spheroid infiltration	EGFR (Cetuximab biosimilar)	AF488	R&D, FAB9577G-100
	CD3	Pacific blue	BioLegend®, 300431
	CD4	PerCP-Cy5.5	BioLegend®, 344608
	CD8	APC-Cy7	BioLegend®, 344714
Block trogocytosis	EGFR (Cetuximab biosimilar)	Biotin	R&D systems, FAB9577B
	Streptavidin	PE	Biolegend®, 405204
	CD3	PE-Cy7	BD Pharmingen™, 557851
	CD4	AF700	BD Pharmingen™, 557922
	CD8	APC-Cy7	BioLegend®, 344714
	MSLN	APC	R&D systems, FAB32652A
	DNA	7AAD	BD Pharmingen™, 51–68981E

The following equation was used to calculate % cytotoxicity: (Experimental value – Effect Cells Spontaneous Control – Target Cells Spontaneous Control)/(Target Cell Maximum Control – Target Cells Spontaneous Control) *100. Specific CAR-mediated lysis was calculated by subtracting the unspecific lysis of target cells by untransduced T cells.

### Spheroid generation and cytotoxicity assays

MSLN^high^GFP^+^ and/or MSLN^low^GFP^+^ SKOV-3 cells were seeded at a total of 5000 cells/well in 96-well ultra-low attachment plates (Corning Costar) in 200 µL cell medium and incubated for 48 hours at 37◦C, 5% CO_2_. Following incubation, the supernatant was removed completely and untransduced, M28z or MBBz transduced T cells were added at a 2:1 effector-to-target ratio. The co-cultures were performed in CellGenix® GMP DC medium (CellGenix) supplemented with 2% human AB serum for 6 or 24 hours. To determine spheroid and supernatant composition, co-cultures were maintained for 6 hours followed by flow cytometry or confocal microscopy (see sections below). Real-time killing of spheroids was monitored over 24 hours of co-culture by addition of CellEvent™ caspase 3/7 green detection reagent (ThermoScientific), followed by detection of caspase activation (green fluorescence) using the IncuCyte S3 live cell imaging system (Essen Bioscience, Sartorius). Analysis was performed with the Incucyte software. To describe the kinetics of cytotoxicity, EC50 values were determined using non-linear curve fit (Graphpad Prism software). The EC50 value represents the timepoint when 50% of the maximum killing (the midpoint of lower and upper plateau) was observed.

### Confocal microscopy

Following 6 hours of co-culture, spheroids were washed, fixed with 4% paraformaldehyde (ThermoScientific), permeabilized in PBS 0,5% Triton-X100 and blocked in PBS 1% BSA (Sigma Aldrich). Primary anti-human CD8a (Biolegend) and anti-human CD4 (R&D Systems) antibodies were added and incubated before DNAHoechst (Invitrogen), secondary rat-anti-mouse AF647 (Biolegend) and donkey-anti-goat AF488 (Thermo Scientific) antibodies were added and incubated for 24 h. For mesothelin staining, spheroids were incubated with PE-conjugated anti-human MSLN antibody (R&D Systems) for 24 h. All stainings were performed in PBS 1% BSA at 4°C. Spheroids were cleared overnight in PBS 88% glycerol (Sigma Aldrich), transferred to ibidi 8-well plates and imaged at the LSM900-airy confocal microscopy. At least three spheroids were imaged per condition, with three images at 10 µm distance per spheroid. Quantification was performed in QuPath.*^25^*

### Fratricide killing assays

HD PBMCs were transduced with the MSLN vector described above and subsequently sorted by FACS to purify the MSLN^high^ and MSLN^low^ T cells. Target T cells were used at three different MSLN frequencies for co-culture: [1] 100% MSLN^high^ cells, [2] 50:50 mixture of MSLN^high^ and MSLN^low^ cells (MSLN^high/low^) and [3] MSLN^low^ target cells alone. Prior to co-culture, all target cells were labeled with CellTrace Violet (CTV, Invitrogen) according to the manufacturer’s protocol. The assay was set up at a 2:1 effector-to-target ratio with a correction performed for the EGFRt% on CAR T cells. Untransduced T cells were included as control at the same number as MSLN-directed CAR T cells. Following four and 24 hours of incubation, cells were harvested and subsequently stained for flow cytometric analysis on the CytoFLEX (Table 1, Fratricide A panel). CAR-mediated lysis of target cells was calculated as follows: $K1a=100\;\times \left(1\;-\left(\frac{CTV^{+\;}7AA{D^{-}}_{CAR}}{CTV^{+\;}7AA{D^{-}}_{UT}}\right)\right)$

In order to investigate fratricide killing of MSLN^+^ CAR T cells in a physiological setting, both MSLN-CAR T cells and MSLN^high^ OVCAR-3 cells were labeled with CTV prior to co-culture at a 1:1 EGFRt^+^ T cell to target cell ratio. Cells were co-cultured for four hours, allowing for trogocytosis to occur. After four hours (t = 0), an equal amount of unlabeled fresh UT, M28z or MBBz CAR T cells were added to the existing co-culture (1:1:1 effector:effector:target ratio) and incubated overnight. The next day, all cells were harvested and subsequently stained for flow cytometric analysis on the CytoFLEX (Table 1, Fratricide B panel). Lysis of MSLN^high^ OVCAR-3 target cells was calculated with formula K1a and fratricide killing of MSLN^+^ M28z or MBBz CAR T cells was calculated as follows:

$$K1b=100\;\times \left(1\;-\left(\frac{CTV^{+\;\;}MSLN^{+\;\;}EGFRt^{+\;\;}7AA{D^{-}}_{+CAR}}{CTV^{+\;\;}MSLN^{+\;\;}EGFRt^{+\;\;}7AA{D^{-}}_{+UT}}\right)\right)$$

Fratricide killing assays were performed at 37◦C and 5% CO_2_ in AimV medium supplemented with 5% HS.

### Functional characterization of trogocytotic CAR T cells

M28z and MBBz CAR T cells were labeled with CTV according to manufacturer’s protocol and co-cultured with MSLN^high^ OVCAR-3 cells at a 1:1 effector-to-target ratio for 1 hour at 37◦C, 5% CO_2_ in AimV medium supplemented with 5% HS. Following incubation, CAR T cells were stained for EGFRt and MSLN as described below and subsequently sorted by FACS (BD FACSAria Fusion, Becton Dickinson) to isolate CTV^+^EGFRt^+^MSLN^+^(/trogo^+^) and CTV^+^EGFRt^+^MSLN^˗^(/trogo^˗^) cells. Purity check post sort was performed on the CytoFLEX flow cytometer. Sorted CAR T cells were counted (t = 0) and cultured for four days in AimV medium supplemented with 5% HS and IL- 2 at 37◦C and 5% CO_2_. On day 1 and 4, CAR T cells were counted and stained for flow cytometric analysis on the CytoFLEX (Table 1, Functional Trogocytosis panel). On day 2, trogo^+^ and trogo^−^ M28z and MBBz CAR T cells were cultured alone (background) or co-cultured with MSLN^high^ OVCAR-3 cells at a 1:1 EGFRt^+^ T cell to target cell ratio on IFNy/TNF/GzB pre-coated fluorospot plates (Mabtech). Following 24 hours of incubation, read-out was performed according to manufacturer’s protocol and spot forming units (SFU) were quantified on the Mabtech IRIS. Co-culture was set up in duplicates. Specific cytokine production was determined as follows: (SFU in CAR + MSLN^high^ OVCAR-3 wells) ˗ (SFU in CAR only wells).

### Bystander killing assays

Killing of MSLN negative target cells was investigated using GFP^+^ K562 cells with differential MSLN expression in Chromium^51^ cytotoxicity assays. K562 cells were labeled with 250 uCi Cr^51^ (Perkin Elmer) per 1E6 cells as previously described. Three different combinations of K562 cells were used: [1] Cr^51^-labeled MSLN negative K562 (ctrl) cells mixed 50:50 with Cr^51^-unlabeled K562-MSLN^+^ cells (ctrl^Cr51+^/MSLN^Cr51-^), [2] Cr^51^-labeled K562 (ctrl^Cr51+^) cells alone and [3] Cr^51^-labeled K562-MSLN^+^ (MSLN^Cr51+^) cells alone. Bystander killing was identified as higher Cr^51^ release in the ctrl^Cr51+^/MSLN^Cr51-^ condition than in the background ctrl^Cr51+^ condition, while MSLN^Cr51+^ were included as a positive control for MSLN-specific lysis.

The co-culture was set up at a 2:1 ratio (CAR to Cr^51^-labeled target cell ratio) in triplicates. Untransduced T cells were included as control at the same number as MSLN-directed CAR T cells. In addition, spontaneous and maximum release wells containing target cells only were included. Following 4 hours of co-culture at 37◦C and 5% CO_2_, 25 µL supernatant was transferred from each well to 200 µL OptiPhase Super Mix in an Isoplate (96-well, Perkin Elmer). The maximum release wells were mixed prior to transfer of 25 µL. Chromium release was detected on a 1450 MicroBeta Liquid Scintillation counter (Perkin Elmer) as counts per minute (CPM). Killing was calculated according to the following:

$K2=100\times \left(\frac{\;\left(\;\frac{CPM_{sample}}{CPM_{spont}}\right)\;}{\left(\;\frac{CPM_{max}}{\;CPM_{spont}}\right)}\right)$ and subsequently normalized as follows: $K2_{CAR}-K2_{UT}$

To assess bystander killing after 24 hours, co-cultures were set up using CTV according to the manufacturer’s protocol for labeling of K562 cells. Three different target cell conditions were included: [1] CTV-labeled MSLN negative control K562 cells mixed 50:50 with Cr^51^-unlabeled MSLN^+^ K562 cells (ctrl^CTV+^/MSLN^CTV-^), [2] CTV-labeled MSLN negative K562 cells (ctrl^CTV+^) alone and [3] CTV-labeled MSLN^+^ K562 (MSLN^CTV+^) cells alone. The assay was performed at a 2:1 ratio (CAR to CTV-labeled target ratio) and set up in duplicates. Untransduced T cells were included as control at the same number as MSLN-directed CAR T cells. Following 24 hours of incubation at 37◦C and 5% CO_2_, cells were harvested and subsequently stained for flow cytometric analysis (CytoFLEX) (Table 1). Target cell lysis was calculated using *K1a*.

Medium used during both bystander killing assays was AimV supplemented with 5% HS and without IL-2

### Blocking of trogocytosis

CAR T cells were pretreated with 1 µM Latrunculin A (Sigma Aldrich) for 15 minutes at 37◦C and 5% CO_2_ prior to co-culture. MSLN^+^GFP^+^ and MSLN^−^GFP^+^ OVCAR-3 cells were subsequently added at a 1:1 effector-to-target ratio. The cells were incubated for 4 hours at 37◦C and 5% CO2, prior to acquisition on the CytoFLEX to measure the level of trogocytosis.

### Flow cytometry analysis

For determining CAR transduction efficiency, M28z and MBBz transduced T cells were first stained for 30 minutes at 4◦C with a primary biotinylated anti-EGFRt/Cetuximab antibody (R&D systems) followed by one wash and subsequent staining with a secondary streptavidin-PE conjugated surface antibody (Biolegend) alongside multiple extracellular antibodies listed in Table 1. To assess the presence of the CAR construct on the T cell surface, cells were first blocked in PBS supplemented with 0,1% bovine serum albumin (BSA, Sigma Aldrich) for 40 minutes at 4◦C and subsequently washed twice, followed by direct staining with a PE-conjugated anti-human Fab (R&D systems) surface antibody together with various other extracellular antibodies (Table 1). Five different extracellular antibody panels were used for characterizing CAR T cells: memory phenotype, phenotypic exhaustion (functional) trogocytosis, fratricide killing, spheroid infiltration, bystander killing, and block-trogocytosis. Cells were incubated with surface antibody mixtures for 30 minutes at 4◦C in the dark. All cell suspensions were resuspended by vortexing following washing and prior to addition of antibodies. Of note: for the spheroid infiltration assay the supernatant was collected separately and spheroids were subsequently dissociated by Trypsin-EDTA (LifeTechnologies). Cell viability was assessed by staining cells with LIVE/DEAD™ Fixable Aqua Dead Cell Stain Kit (Invitrogen) according to manufacturer’s instruction or by staining cells with 7AAD for 7 minutes at room temperature after completion of surface staining (Table 1). To assess both CAR T cell and target cell phenotype following co-culture, non-adherent cells were harvested by aspiration and remaining adherent cells were harvested by trypsinization (Hyclone Trypsin Protease, Cytiva). All cells were collected in the same collection tube and stained simultaneously.

Intracellular cytokine staining (ICS) was performed as reported previously by co-culturing target cells with CAR T cells in the presence of brefeldin A (BFA, Sigma-Aldrich), Golgi-Stop (BD Biosciences) and anti-CD107a-PE (BD Pharmingen) for 6 hours.*^23^*

Tumor cells were checked weekly by flow cytometry for the presence of surface MSLN (R&D systems) and GFP signal.

Cells were acquired using FACS Canto (Becton Dickinson), CytoFLEX (Beckman Coulter) or NovoCyte3000 (ACEA Biosciences) and subsequent data output was analyzed with FlowJo software (FlowJo).

### Statistics

Graphpad Prism software was used to perform data analysis and visualization. To determine the highest Caspase 3/7 signal, R software was used. Data were represented as median, except the Caspase 3/7 signal overtime which was displayed as mean+SEM. For confocal microscopy and Incucyte assays, three technical replicates were included in each experiment. All experiments were independently repeated with 3–10 different donors (biological replicates). Wilcoxon matched pairs signed rank test was used to compare two groups of paired samples. Friedman tests followed by Dunn’s multiple comparison tests were performed to compare ≥3 paired samples. Correlation between two factors was assessed by linear regression (R^2^) and/or Spearman correlation (R). Unbiased hierarchical clustering was performed with online CIMminer, output was a two-dimensional clustering image map using the Euclidean distance method and average linkage cluster algorithm.

## Results

### Phenotypic and functional differences between M28z and MBBz CAR T cells

MSLN-CAR transduction efficiency, as detected by EGFRt expression, was assessed four and eight days following retroviral exposure. Transduction efficiency was comparable between M28z- and MBBz-transduced T cells on day 4 (sup. Figure 1a), however, the frequency of M28z-transduced T cells increased significantly between day 4 and day 8. The median transduction efficiency on day 8 was 77% and 66% in M28z- and MBBz-transduced T cells, respectively, resulting in MSLN-CAR T cell products composed of transduced (CAR^+^) and non-transduced (CAR^˗^) T cells (Sup. Figure 1a). Eight days after transduction with either the M28z or MBBz CAR construct, the CAR T cell phenotype was determined by flow cytometric analysis:

**Figure 1.**
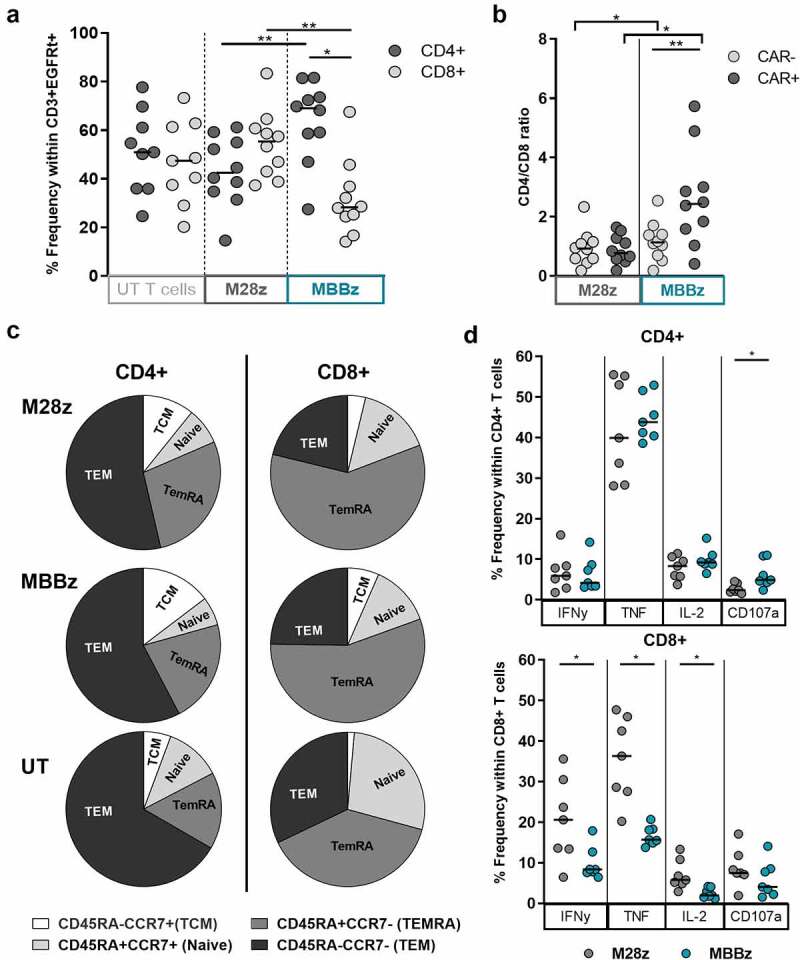
**Phenotypic characterization of MSLN-CAR T cells after CAR production**. A) Frequency of CD4^+^ and CD8^+^ cells within CD3^+^EGFRt^+^ M28z and MBBz CAR T cells and within CD3^+^ untransduced T cells (UT). N = 10 donors. B) Ratio of CD4^+^/CD8^+^ within EGFRt^+^ (CAR^+^) and EGFRt- (CAR^−^) M28z and MBBz transduced T cells. N = 10 donors. C) Maturation phenotype determined by CD45RA and CCR7 expression in CD3^+^EGFRt^+^ M28z and MBBz CAR T cells and within CD3^+^ UT cells. Gated on lymphocytes→ single cells→ viable cells (7AAD^−^)→ CD3^+^EGFRt^+^ (M28z and MBBz) or CD3^+^EGFRt^−^ (UT)→ CD4^+^/CD8^+^ and ultimately CD45RA/CCR7. Median of n = 9 donors displayed. D) IFNy, TNF, IL2 cytokine production and CD107a degranulation by CD4^+^ and CD8^+^ M28z and MBBz transduced T cells following 6 hours of co-culture with OVCAR-3 MSLN^+^, determined by intracellular cytokine staining. Grey symbols represent M28z CAR T cells and green symbols represent MBBz CAR T cells. Wilcoxon tests were performed to detect differences between and within M28z and MBBz CAR constructs. For comparison of three T cell subsets (M28z vs MBBz vs UT) Friedman tests were used. * = P < .05 and ** = P < .01. Each dot represents 1 donor.

We quantified the CD4 and CD8 T cell frequency in CAR^+^ and CAR^˗^ fractions of MSLN-CAR transduced T cells as well as in control untransduced T cells (UT). The frequency of CD8^+^ T cells was significantly higher within M28z CAR^+^ T cells as compared to MBBz CAR^+^ T cells. Accordingly, the CD4^+^ population was predominant within MBBz CAR^+^ T cells (Figure 1a). The bias toward the CD4^+^ population was specific for the CAR^+^ fraction as compared to the CAR˗ fraction of MBBz transduced T cells, while the CD4^+^/CD8^+^ ratio was comparable between the M28z CAR^+^ and CAR^˗^ T cells (Figure 1b).

Differentiation phenotype was comparable between M28z and MBBz CAR^+^ T cells (Supp. Figure 1b). Compared to UT cells, the terminally differentiated effector memory T cell (TemRA, CCR7^−^CD45RA^+^) population was significantly elevated in M28z CD4^+^ and CD8^+^ CAR^+^ T cells (Figure 1c and sup. Figure 1a). Whereas central memory T cell (TCM, CCR7^+^CD45RA^−^) frequencies were significantly higher in MBBz CD4^+^ and CD8^+^ CAR^+^ T cells as compared to UT cells (Sup. Figure 1b).

To ensure stable and high MSLN expression by target cells in functional assays, OVCAR-3, SKOV-3 and K562 cells were transduced with MSLN, resulting in MSLN^high^ target cells while parental target cells expressed low MSLN surface levels (Sup. Material 1A-B). In response to co-culture with MSLN^high^ OVCAR-3 target cells, M28z- and MBBz-transduced CD4 + T cells produced comparable levels of IFNy, TNF, and IL-2, while CD107 expression was significantly increased in MBBz-transduced CD4^+^ T cells compared to their M28z counterparts. In contrast, differences were observed in the CD8^+^ T cell subset with increased IFNy, TNF, and IL-2 production by M28z-transduced T cells as compared to MBBz-transduced T cells (Figure 1d). Co-cultures performed with MSLN^high^ K562 target cells revealed comparable trends (Sup. Figure 1c).

### Superior lysis of MSLN^high^ SKOV-3 spheroids by M28z CAR T cells

To determine whether the cytolytic capacity is affected by the CD4^+^ bias and the reduced cytokine production by CD8^+^ CAR T cells following MBBz transduction, CD4^+^ and CD8^+^ CAR T cells were sorted prior to cytotoxicity assays.

**Figure 2.**
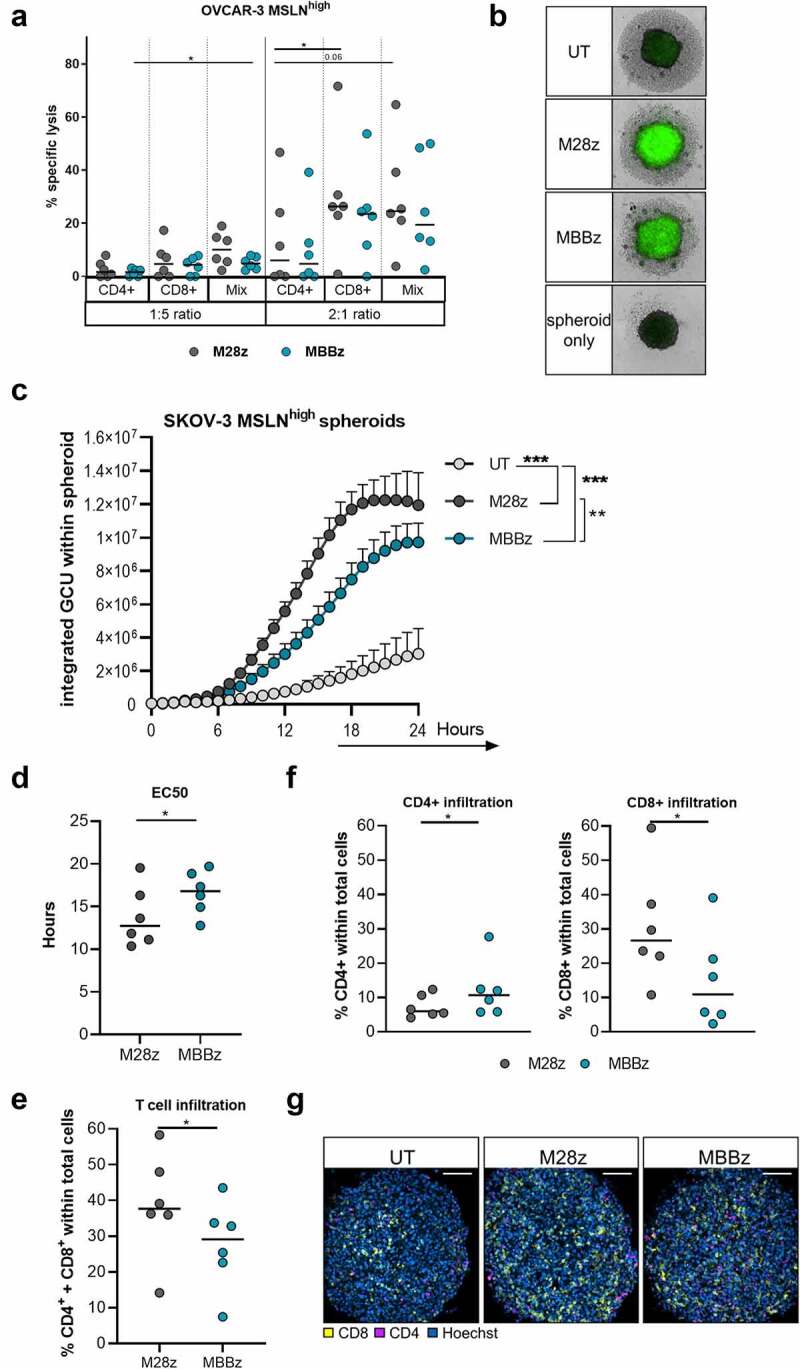
**Superior lysis and infiltration of MSLN^high^ SKOV-3 spheroids by M28z CAR T cells as compared to MBBz CAR T cells**. A) Lysis of MSLN^high^ OVCAR-3 cells as determined by LDH release following co-culture with CD4^+^ enriched, CD8^+^ enriched and unsorted CD4^+^/CD8^+^ MSLN-CAR T cells at a 1:5 or 2:1 effector to target ratio for 24 hours. N = 6 donors, each dot represents one donor. B, C) Lysis of MSLN^high^ SKOV-3 spheroids in response to co-culture with UT cells, M28z or MBBz transduced T cell was monitored for 24 hours by detection of Caspase3/7 activation using the IncuCyte S3 live cell imaging system. B) Caspase3/7 activation as depicted by the green signal in representative spheroid. C) Caspase3/7 activation illustrated by the integrated GCU over time. Dots represent the mean+SEM of 3 technical replicates of 6 donors. GCU = green calibrated unit. D) Non-linear regression revealed EC50 of Caspase3/7 activation. E) Frequency of total T cells (%CD4^+^+CD8^+^) within SKOV-3 MSLN^high^ spheroids as detected by confocal microscopy following 6 hours of co-culture. F) CD4^+^ or CD8^+^ T cells within MSLN^high^ SKOV-3 spheroids as detected by confocal microscopy after 6 hours of co-culture. Each dot represents the mean of 3 technical replicates from 1 donor, n = 6 donors. G) Representative confocal image of T cell infiltration of one donor. Grey symbols represent M28z CAR T cells and green symbols represent MBBz CAR T cells. Friedman tests were performed to detect differences in target cell lysis by CD4^+^, CD8^+^ or CD4^+^/CD8^+^ T cells (OVCAR-3 vs SKOV-3). Wilcoxon was performed to compare OVCAR-3 vs SKOV-3 per T cell condition and M28z vs MBBz. One-way ANOVA was used to compare caspase3/7 signal over a 24 hour time course in response to UT, M28z or MBBz treatment. N = 6. * = P < .05, ** = P < .01, *** = P < .001.

The CD4^+^ enriched, CD8^+^ enriched, or unsorted (CD4^+^/CD8^+^ mixed) M28z or MBBz CAR T cells were co-cultured for 24 hours with MSLN^high^ OVCAR-3 or SKOV-3 target cells. Sorted CD8^+^ and unsorted CD4^+^/CD8^+^ MSLN-CAR T cell mediated killing of MSLN^high^ OVCAR-3 cells was significantly elevated compared to sorted CD4^+^ M28z and MBBz CAR T cells (Figure 2a). This indicates that killing of MSLN^high^ OVCAR-3 cells was CAR T cell subset-dependent. The median lysis of OVCAR-3 by CD4^+^ enriched CAR T cells was similar at the 1:5 and 2:1 ratio, indicating that increasing the number of CD4^+^ CAR T cells alone will not enhance killing of this particular cell line. No differences in lysis of MSLN^high^ SKOV-3 cells by sorted CD4^+^, sorted CD8^+^ and unsorted CD4^+^/CD8^+^ M28z and MBBz CAR T cells were detected (Sup. Figure 2a).

To further investigate whether differences in killing capacity between M28z and MBBz CAR T cells can be detected *in vitro*, experiments using real-time visualization of cell killing (IncuCyte®) and confocal microscopy were performed. Untransduced T cells, M28z, and MBBz CAR T cells were co-cultured with MSLN^high^ SKOV-3 spheroids and real-time lysis of the spheroid grown tumor cells was assessed by detection of Caspase3/7 activation during 24 hours (Figure 2b). Both M28z and MBBz CAR T cells displayed superior cytolytic capacity against MSLN^high^ SKOV-3 spheroids, as compared to untransduced T cells (Figure 2c). However, M28z CAR T cells were more efficient at spheroid grown tumor cell lysis as indicated by the overall higher caspase activation as well as the significant difference in time to EC50 in M28z (median EC50 = 12,74 hours) as compared to MBBz (median EC50 = 16,80 hours) (Figure 2b-d, Sup. Figure 2b).

Furthermore, infiltration of MSLN^high^ SKOV-3 spheroids by CD4^+^ and CD8^+^ T cells was assessed by confocal microscopy and flow cytometry. Whole spheroids were used for imaging, allowing for quantification of infiltrating CD4/CD8 lymphocytes (Figure 2e-f). M28z transduced T cells displayed superior spheroid infiltration as compared to MBBz transduced T cells (Figure 2e). Prior to start of co-culture, the EGFRt% was comparable between M28z and MBBz CAR T cells, and MBBz CAR T cells showed a bias toward the CD4^+^ population, while M28z CAR T cells displayed a higher frequency of CD8^+^ T cells (Sup. Figure 2c-d). In line with starting frequencies, quantitatively more CD8^+^ lymphocytes were detected in spheroids co-cultured with M28z CAR T cells, whereas higher frequencies of CD4^+^ infiltrating lymphocytes were found within MBBz-treated spheroids (figure 2f-g). Flow cytometric analysis of the CAR T cell-infiltrated (spheroids) and non-infiltrated (supernatant) cell fractions revealed accumulation of CD4^+^ T cells in the supernatant and enrichment of CD8^+^ T cells within spheroids for both M28z and MBBz transduced T cells (Sup. Figure 2e). These results indicate CD8^+^ CAR T cells harbor superior tumor spheroid-infiltrating capacity compared to CD4^+^ CAR T cells.

### M28z CAR T cells efficiently target and lyse MSLN^low^ target cells

**Figure 3.**
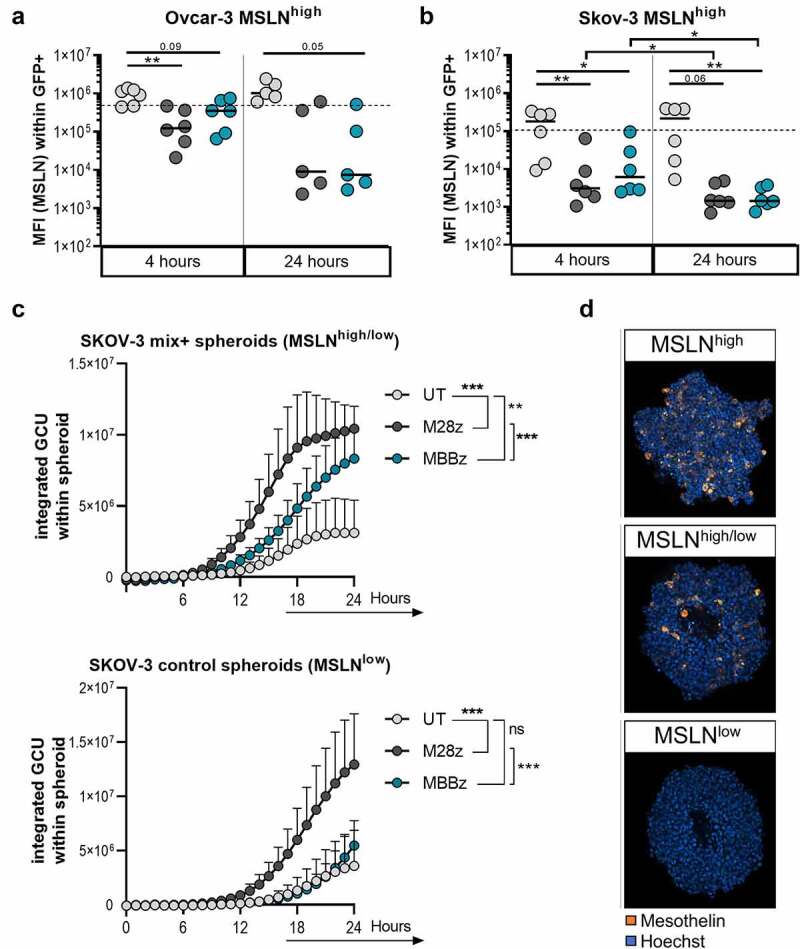
**CAR-mediated loss of MSLN surface expression by target cells and efficient lysis of MSLN^low^ target cells by M28z CAR T cells**. MFI of MSLN (logarithmic y-axis) within GFP^+^ A) OVCAR-3 MSLN^high^ and B) SKOV-3 MSLN^high^ target cells, as detected by GFP^+^ signal through flow-cytometric analysis. Dotted line represents MFI of MSLN within GFP^+^ target cells upon start of co-culture (median of 6 individual experiments). N = 6 donors, each dot represents one donor. C) Spheroids were generated with control SKOV-3 cells (MSLN^low^) alone and a 50:50 mix of MSLN^high^ and control MSLN- SKOV-3 cells (MSLN^high/low^). Co-cultures were set up a 2:1 ratio with UT cells, M28z or MBBz transduced T cells. Lysis was monitored by detection of Caspase3/7 activation for 24 hours. GCU = green calibrated unit. Dots represent the mean+SEM of 3 technical replicates of 6 donors. D) Representative images of MSLN staining of the three variations of SKOV-3 spheroids used (MSLN^high^, MSLN^high/low^ and MSLN^low^). Dark gray symbols represent M28z CAR T cells, green symbols represent MBBz CAR T cells and light gray represents UT cells. Friedman tests were performed to compare MSLN expression on target cells in response to different T cell treatments. One-way ANOVA was used to compare caspase3/7 signal over a 24 hour time course in response to UT, M28z or MBBz treatment. Wilcoxon tests were performed to compare between 4 hours and 24 hours. n = 6. * = P < .05, ** = P < .01, *** = P < .001.

During co-culture with MSLN-CAR T cells, a reduction in MSLN surface expression on MSLN^high^ OVCAR-3 and SKOV-3 target cells was observed, as determined by MSLN median fluorescence intensity (MFI) and frequency within GFP^+^ target cells (Figure 3a-b). Of note, MSLN surface expression was significantly higher on MSLN-transduced OVCAR-3 than SKOV-3 cells, with a median of 95% and 78%, respectively (Sup. Figure 3a-b). The gradual overtime loss (4 h vs. 24 h co-culture) in MSLN surface expression on OVCAR-3 and SKOV-3 target cells was specific for co-cultures with M28z and MBBz CAR T cells, and not UT cells, suggesting the loss was CAR-mediated (Figure 3a-b, Sup. Figure 3b-c). Yet, the reduction in the frequency of MSLN^+^ tumor cells was only significant between the 4 hour and 24 hour time point for MSLN^high^ SKOV-3 cells (Figure 3b). Following 24 hours of exposure to either M28z or MBBz CAR T cells, median MSLN surface expression levels decreased from 95% to 42–51% and from 78% to 10–12% on MSLN^high^ OVCAR-3 and SKOV-3 cells, respectively (Sup. Figure 3a-c).

Due to the significant reduction in MSLN surface expression on MSLN^high^ OVCAR-3 and SKOV-3 cells, we aimed to determine whether target cell lysis was affected by lower frequencies of MSLN^+^ target cells. To generate SKOV-3 spheroids with heterogeneous MSLN expression (MSLN^high/low^), parental SKOV-3 cells (MSLN^low^) and transduced MSLN^high^ SKOV-3 cells were mixed 50:50. Detection of MSLN by confocal microscopy confirmed a range of MSLN expression in MSLN^high^, MSLN^high/low^ and MSLN^low^ spheroids (Figure 3d). Notably, M28z and MBBz MSLN specific CAR T cells demonstrated significantly increased lysis of the heterogeneous MSLN^high/low^ spheroids relative to untransduced T cells (Figure 3c). However, the M28z CAR T cells showed superior lysis capacity of the heterogeneous MSLN^+^ spheroids compared to MBBz CAR T cells (Figure 3c). Furthermore, M28z CAR T cells were efficient in targeting and lysing parental MSLN^low^ tumor spheroids, while MBBz CAR T cells did not mediate CAR-specific lysis in this low antigen stimulation setting. Regardless of MSLN frequency on SKOV-3 spheroids, M28z showed superior lysis compared to both MBBz CAR T cells and untransduced T cells (Figure 3d, Sup. Figure 3d).

To investigate whether lysis of the parental OVCAR-3 and SKOV-3 cells could occur independently of CAR-MSLN antigen interaction, a mechanism referred to as ‘bystander killing’, killing of parental K562 cells (ctrl, MSLN devoid) and K562 cells transduced with MSLN was measured using chromium^51^ (Cr^51^) assays (Sup. Figure 3e). Bystander killing was identified as higher Cr^51^ release in the ctrl^Cr51+^/MSLN^Cr51-^ condition than in the background ctrl^Cr51+^ condition. No bystander killing was observed following 4 hours of co-culture with either M28z and MBBz CAR T cells (Sup. figure 3f). M28z and MBBz CAR T cells were capable of lysing MSLN^+^ K562 cells, with elevated chromium release in the MSLN^Cr51+^ condition relative to the ctrl^Cr51+^conditions. These results were confirmed by flow cytometry using CellTrace Violet (CTV) labeled K562 cells instead of Cr^51^-labeling, with comparably low lysis of ctrl^CTV+^ and ctrl^CTV+^/MSLN^CTV-^ cells by M28z and MBBz CAR T cells following 24 hours of co-culture (Sup. Figure 3g).

### M28z and MBBz CAR T cells display trogocytotic capacity

**Figure 4.**
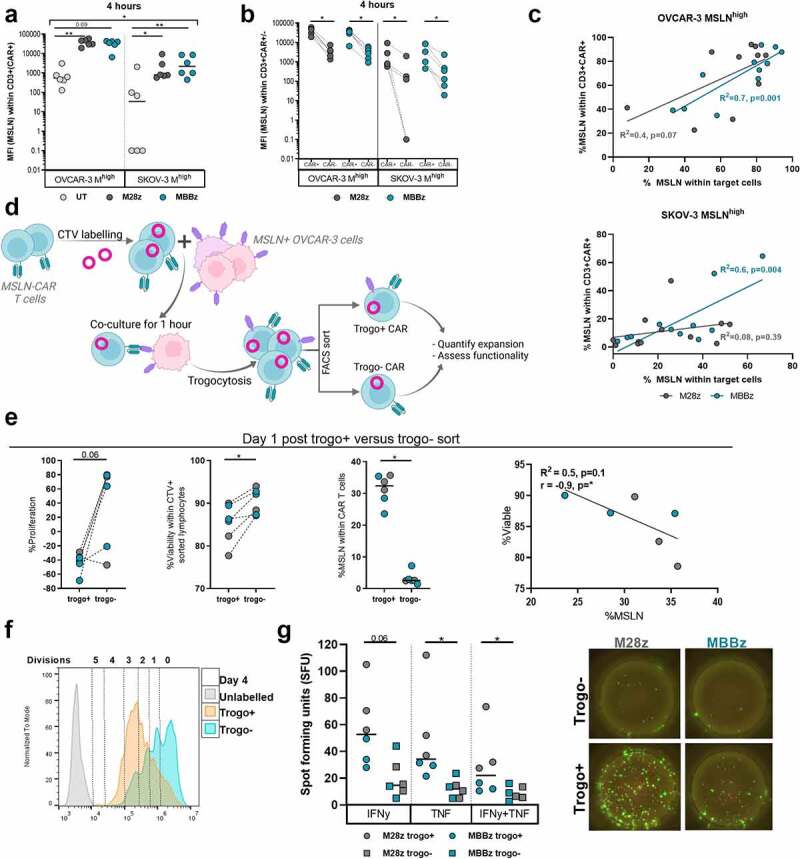
**Trogocytosis of MSLN antigen by M28z and MBBz CAR T cells**. A) MFI of MSLN (logarithmic y-axis) within CD3^+^CAR^+^ M28z and MBBz CAR T cells or CD3^+^CAR^−^ UT cells following 4 hours of co-culture with MSLN^high^ OVCAR-3 and SKOV-3 cells. B) MFI of MSLN (logarithmic y-axis) within CAR^+^ and CAR^−^ M28z and MBBz transduced T cells. CAR expression was determined by hFAB staining. C) Linear regression between MSLN expression on target cells and CAR T cells. N = 6 donors, each dot represents one donor. D) Graphical outlay of experimental set-up for sorting trogo^+^ and trogo^˗^ CAR T cells. CAR T cells were sorted on CTV^+^EGFRt^+^MSLN^+^(/trogo^+^) or CTV^+^EGFRt^+^MSLN^−^(/trogo^˗^). Following sort, trogo^+^ and trogo^˗^ cells were counted (t = 0) and subsequently cultured for four more days. E) On day 1, CAR T cells were counted and % proliferation compared to t = 0 was calculated (left) Furthermore, viability and MSLN frequency within trogo^+^ and ^˗^ was quantified by flow cytometry (middle). Linear regression between viability and MSLN frequency within trogo^+^ CAR T cells (right). Each dot represents M28z or MBBz CAR T cells from 1 donor, n = 3 donors. F) Representative overlay of CTV dilution four days post sort. 1 out of 3 donors is displayed. G) Specific IFNy and TNF spot forming units (SFU) by trogo^+^ and trogo^˗^ CAR T cells following background normalization (left). Each dot represents M28z or MBBz CAR T cells from 1 donor, n = 3 donors. F) Representative fluorospot wells displaying IFNy (green) and TNF (pink) production by trogo^+^ and trogo^−^ CAR T cells. 1 out of 3 donors is shown. Dark gray symbols represent M28z CAR T cells, green symbols represent MBBz CAR T cells and light gray represents UT cells. Friedman tests were performed for comparison between T cells (UT vs M28z vs MBBz). Wilcoxon tests were used to compare within one CAR construct. * = P < .05, ** = P < .01.

To investigate the mechanism behind the loss of MSLN cell surface expression on MSLN^high^ OVCAR-3 and SKOV-3 cells during co-culture with M28z and MBBz CAR T cells (Figure 3a-b), CAR-mediated trogocytosis was assessed by flow cytometry (Sup. Figure 4a). Notably, 4 hours after start of co-culture, M28z, and MBBz CAR T cells had already acquired MSLN expression and displayed substantially elevated levels of MSLN compared to untransduced T cells, as indicated by the elevated MSLN MFI and frequency within (CAR) T cells (Figure 4a, Sup. Figure 4b). Interestingly, the frequency of MSLN^+^ CAR T cells was significantly higher during co-cultures with MSLN^high^ OVCAR-3 than MSLN^high^ SKOV-3 cells. Furthermore, MSLN expression was significantly higher within CAR^+^ than CAR^˗^ M28z- and MBBz-transduced T cells, indicating CAR-mediated trogocytosis (Figure 4b, Sup. Figure 4c). One prerequisite for trogocytosis is antigen availability, and in accordance with this, M28z and MBBz CAR T cells displayed higher levels of MSLN upon co-culture with MSLN^high^ OVCAR-3 and SKOV-3 cells as compared to their respective parental MSLN^low^ cell lines (Sup. Figure 4d). Of note, low frequencies of M28z and MBBz CAR T cells presenting surface expression of MSLN were detected in co-cultures with parental OVCAR-3 and SKOV-3 cells, suggesting the presence of MSLN^+^ parental cells, albeit a minimal one (Sup. Fig. 4a and 5c). Linear regression analysis revealed a strong correlation between MSLN^+^ MBBz CAR T cells and MSLN frequency on transduced MSLN^high^ OVCAR-3 and SKOV-3 cells (Figure 4c). The same trend was observed for M28z CAR T cells during co-culture with MSLN^high^ OVCAR-3 cells. However, no correlation was found between MSLN^+^ M28z CAR T cells and MSLN frequency on transduced SKOV-3 cells (Figure 4c). The frequency of MSLN^+^ M28z and MBBz CAR T cells was substantially higher following 4 hours of co-culture with MSLN^high^ OVCAR-3 and SKOV-3 as compared to 24 hours (Sup. Figure 4e). Noteworthy, CD4^+^ CAR T cells displayed higher levels of MSLN expression compared to their CD8^+^ counterparts, suggesting enhanced trogocytotic capacity by CD4^+^ MSLN-CAR T cells. To determine the effect of trogocytosis on CAR T cell proliferation and functionality, M28z and MBBz CAR T cells were sorted based on MSLN expression (trogo^+^ versus trogo^−^) following brief exposure to MSLN^high^ OVCAR-3 cells (Figure 4d). The freshly isolated MSLN^+^(/trogo^+^) or MSLN^˗^(/trogo^˗^) CTV^+^EGFRt^+^ CAR T cells were counted, and cultured for four days. The absolute number of viable trogo^+^ CAR T cells decreased overnight, as indicated by median proliferation of ˗40%, while the trogo^˗^ CAR T cells were able to proliferate overnight (median proliferation of 71%) (Figure 4e). In line with this, the viability of trogo^+^ CAR T cells was significantly reduced as compared to ^˗^ CAR T cells. Trogo^+^ sorted CAR T cells maintained MSLN surface expression to a certain degree following overnight incubation, however, the frequency of MSLN^+^ CAR T cells diminished during four days of culture (Figure 4e and Sup. figure 4f). Notably, the viability of trogo^+^ sorted CAR T cells negatively correlated with MSLN surface expression levels. Taken together, these data are indicative of fratricide killing of MSLN^+^ CAR T cells.

**Figure 5.**
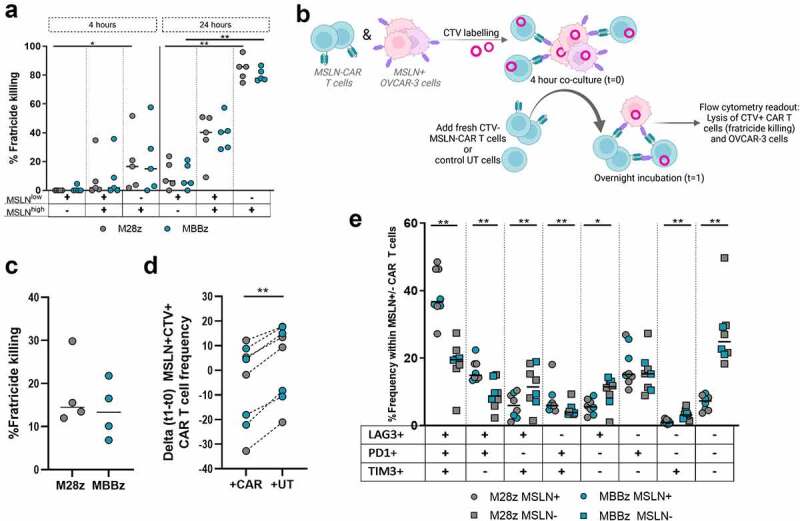
**Fratricide killing of MSLN^+^ M28z and MBBz CAR T cells**. A) M28z and MBBz CAR T cells were co-cultured with MSLN^high^, MSLN^low^ and mixed MSLN^high/ow^ autologous T cells for 4 and 24 hours at a 2:1 ratio. CAR-mediated fratricide killing of autologous T cells is displayed following normalization with UT cells. Each dot represents one donor, n = 5 donors. B) Graphical illustration for assessment of fratricide killing during co-culture with MSLN^high^ OVCAR-3 cells. C) Fratricide killing of CTV^+^EGFRt^+^MSLN^+^ CAR T cells after normalization with UT cells. D) Delta frequency of CTV^+^EGFRt^+^MSLN^+^ CAR T cells following overnight incubation with freshly added CAR T cells or UT cells (delta t1-t0). E) PD-1/LAG-3/TIM-3 (co-)expression within trogocytotic MSLN^+^ (circles) or MSLN^˗^ (squares) CTV^+^EGFRt^+^ CAR T cells following overnight co-culture with MSLN^high^ OVCAR-3 cells and CTV^˗^ CAR T cells. Each dot represents M28z or MBBz CAR T cells from one donor, n = 4 donors. Grey symbols represent M28z CAR T cells and green symbols represent MBBz CAR T cells. Friedman tests were performed for comparison between target cells (MSLN^high^ vs MSLN^high/low^ vs MSLN^low^). Wilcoxon tests were used to compare within and between CAR constructs. * = P < .05, ** = P < .01.

Despite the initial decline in proliferation, trogo^+^ CAR T cells proliferated significantly more than trogo^˗^ CAR T cells (figure 4f and Sup. Figure 4g). Furthermore, trogo^+^ CAR T cells displayed superior IFNy and TNF production compared to trogo^˗^ CAR T cells following 24 hours of co-culture with MSLN^high^ OVCAR-3 CAR T cells (Figure 4g).

To confirm if trogocytosis is indeed a driving force behind the high levels of MSLN expression by M28z and MBBz CAR T cells when incubated with MSLN^high^ target cells, blocking experiments using Latrunculin A (LatA) were performed. The intercellular transfer of membrane and membrane-bound molecules during trogocytosis requires actin remodeling and LatA disrupts this microfilament polymerization. Pre-treatment of MSLN^+^ OVCAR-3 with LatA before co-culture induced 50% reduction of the frequency of MSLN^+^ M28z and MBBz CAR T cells indicating trogocytosis-mediated MSLN transfer between CAR T cells and target cells (Sup. Figure 4h). Again, as seen in Figure 4b using direct CAR staining (hFAB), trogocytosis was higher in M28z and MBBz transduced (EGFRt+) CAR T cells as compared to non-transduced T cells.

### Fratricide killing of MSLN^+^ CAR T cells by M28z and MBBz CAR T cells

The expression of MSLN by M28z and MBBz CAR T cells after trogocytosis poses a risk for MSLN-directed CAR T cell lysis of CAR T cells expressing MSLN, in other words ‘fratricide killing’. Fratricide killing may explain the reduced proliferation and viability in trogo^+^ as compared to trogo^˗^ sorted CAR T cells following overnight incubation observed previously (Figure 4e). To investigate this possibility, T cells were transduced with MSLN and subsequently sorted to isolate MSLN^low^ and MSLN^high^ target cells. M28z and MBBz CAR T cells were co-cultured with matched donor target T cells expressing MSLN at three different frequencies: MSLN^low^ cells, 50:50 mixed MSLN^low^ and MSLN^high^ cells (MSLN^high/low^) and MSLN^high^ cells (Sup. Figure 5a). CAR-mediated fratricide killing of MSLN^high^ T cells was detected already at 4 h of co-culture and maximum lysis was reached following 24 hours of co-culture with M28z and MBBz CAR T cells (normalized median lysis of 86% and 78%, respectively, Figure 5a). The degree of fratricide killing was significantly enhanced in MSLN^high^ T cells compared to MSLN^low^ T cells. As such, the frequency of MSLN^+^ T cells at the start of co-culture was predictive of the degree of fratricide killing by CAR T cells after 24 hours. Trogocytosis of MSLN by M28z and MBBz CAR T cells from MSLN^high/low^ and MSLN^high^ T cells was observed during fratricide cultures, albeit minimally, with frequency of MSLN^+^ CAR T cells ranging between median 4–9% following 4 hours of co-culture and 1–2% after 24 hours (Sup. Figure 5b).

To confirm these findings in a physiological setting, MSLN^high^ OVCAR-3 and MSLN-CAR T cells were both labeled with CTV and co-cultured for 4 hours, allowing for trogocytosis to occur. Following 4 hours of co-culture, fresh CTV-unlabeled UT or CAR T cells were added in order to assess lysis of MSLN^+^CTV^+^ CAR T and MSLN^high^ OVCAR-3 cells (Figure 5b). Freshly added M28z and MBBz CAR T cells performed fratricide killing of MSLN^+^CTV^+^ CAR T cells, with median fratricide killing of 15% and 13%, respectively (Figure 5c). This was further confirmed by the reduced frequency of MSLN^+^CTV^+^ CAR T cells following overnight incubation (t1-t0) in response to addition of CTV^˗^ M28z or MBBz CAR T cells as compared to UT cells (Figure 5d). Of note, these graphs merely display the additive fratricide killing effect exerted by CTV^˗^ CAR T cells and do not reflect fratricide killing by CTV^+^ CAR T cells. Moreover, the freshly added CAR T cells aided in MSLN^high^ OVCAR-3 lysis as compared to UT cells, suggesting CTV^˗^ CAR T cells mediated tumor cell lysis as well as fratricide killing (Sup. Fig. C-D). As expected, CTV^˗^ M28z and MBBz CAR T cells also displayed trogocytotic capacity (Sup. Figure 5e). Taken together, these results demonstrate the dynamic process of trogocytosis and subsequent fratricide killing by M28z and MBBz CAR T cells.

Trogocytotic MSLN^+^ CAR T cells displayed higher (co-)expression levels of the PD-1, LAG-3 and/or TIM-3 co-inhibitory molecules (CIMs) as compared to MSLN^˗^ CAR T cells (Figure 5e). The frequency of PD-1/LAG-3/TIM-3 triple positive (TP) cells was significantly increased in MSLN^+^ M28z and MBBz CAR T cells, while the frequency of PD-1/LAG-3/TIM-3 triple negative (TN) cells was substantially higher in MSLN^˗^ M28z and MBBz CAR T cells, suggesting trogocytotic CAR T cells were more exhausted than their non-trogocytotic counterparts.

### Dynamic expression of PD-1, LAG-3, and TIM-3 by M28z and MBBz CAR T cells

**Figure 6.**
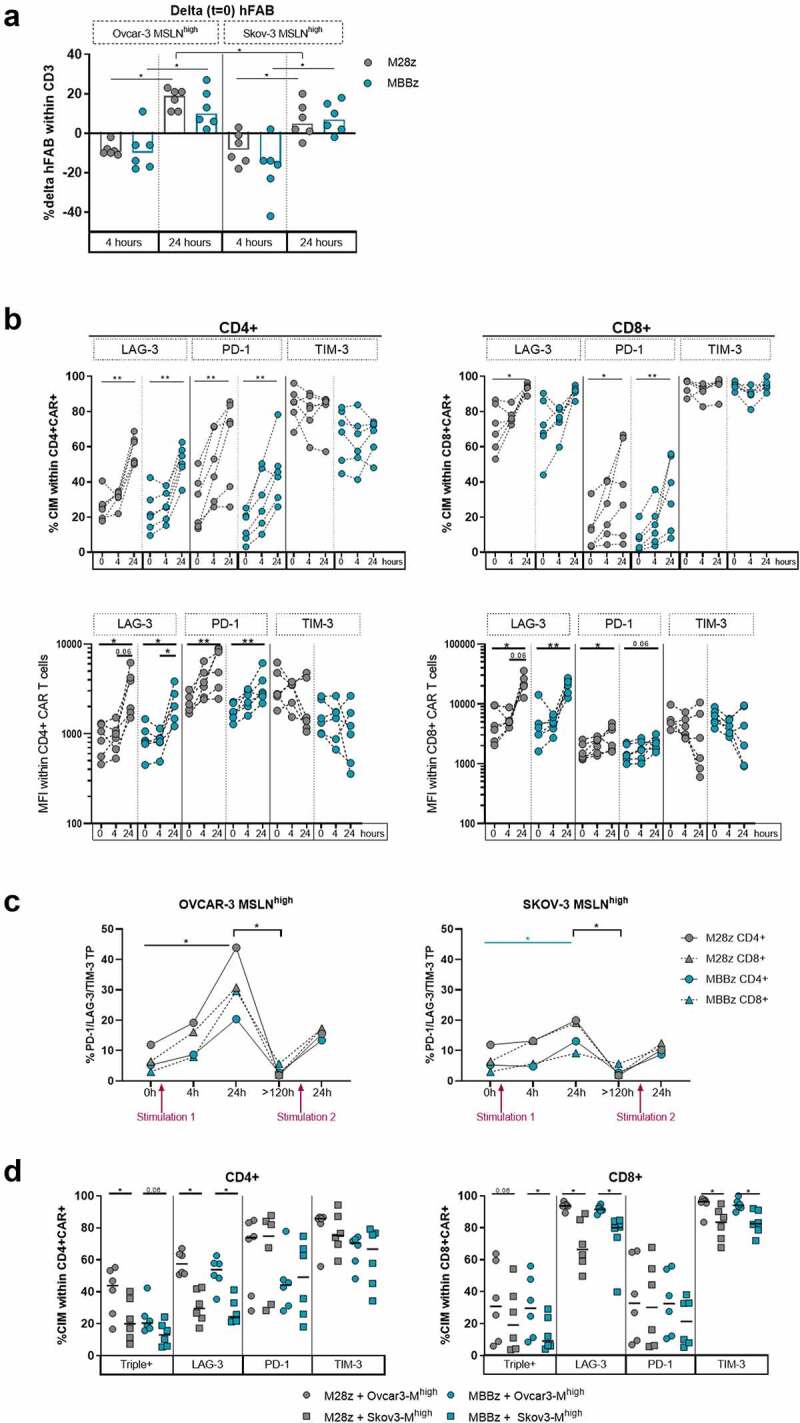
**Dynamic expression of PD-1, LAG-3 and TIM-3 exhaustion markers by M28z and MBBz CAR T cells after co-culture with target cells**. A) Prior to start of experiment (t = 0), CAR surface expression on M28z and MBBz transduced T cells was determined by hFAB staining. During co-culture with MSLN^high^ OVCAR-3 and SKOV-3 hFAB expression was monitored. B) Kinetics of PD-1, LAG-3 and TIM-3 frequency (top) and MFI (bottom, logarithmic y-axis) within CD4^+^ or CD8^+^ CAR^+^ M28z and MBBz transduced T cells prior (0 h) and during co-culture (4 h and 24 h) with MSLN^high^ OVCAR-3 cells. Each dot represents one donor, n = 6 donors. C) Frequency of PD-1/LAG-3/TIM-3 triple positive (TP or Triple+) within CAR^+^ M28z and MBBz transduced T cells overtime. Median of 6 donors is displayed. D) Comparison between CIM (co-)expression following 24 hours of co-culture with MSLN^high^ OVCAR-3 and SKOV-3 cells. Each dot represents one donor, n = 6 donors. Grey symbols represent M28z CAR T cells and green symbols represent MBBz CAR T cells. Wilcoxon tests were performed to compare between two time points, CAR constructs and cell lines (delta hFAB 4 hours vs 24 hours, M28z vs MBBz, and OVCAR-3 vs SKOV-3). Friedman tests were used per CAR construct over time (≥3 time points). * = P < .05, ** = P < .01.

We investigated the cell surface phenotype of the M28z and MBBz CAR T cells during co-culture with MSLN^high^ OVCAR-3 and SKOV-3 cells by measuring expression levels of the MSLN-CAR construct (as determined by hFAB) and the PD-1, LAG-3, and TIM-3 CIMs overtime by flow cytometry. Following 4 hours of exposure to MSLN^high^ OVCAR-3 and SKOV-3 cells, hFAB surface expression decreased relative to the original expression (Figure 6a) (median hFAB% at start was 52% for M28z and 57% for MBBz). After 24 h of exposure to MSLN^high^ target cells, hFAB surface expression increased substantially more following exposure to OVCAR-3 compared to SKOV-3 cells (around 20% above original expression) (Figure 5a). Furthermore, during co-culture, PD-1, LAG-3, and TIM-3 expression levels were dynamic in M28z and MBBz CAR T cells, as determined by both frequency and MFI (Figure 6b and Sup. Figure 6a). PD-1 expression on CD4^+^ and CD8^+^ M28z and MBBz CAR T cells increased significantly over 24 hours of co-culture with both MSLN^high^ OVCAR-3 and SKOV-3, while LAG-3 expression only increased following exposure to MSLN^high^ OVCAR-3 cells (Figure 6b and Sup. Figure 6a). Frequency of TIM-3^+^ cells was remarkably high at the start of co-culture in both CD4^+^ and CD8^+^ CAR T cell populations (Figure 6b). Notably, TIM-3 frequency and MFI within M28z and MBBz CAR T cells decreased significantly during 4 hours of co-culture with MSLN^high^ SKOV-3, while TIM-3 expression remained consistently high throughout co-culture with MSLN^high^ OVCAR-3 cells (Figure 6b and Sup. Fig, 6A).

We then focused on the single and combinatorial PD-1, LAG-3 and/or TIM-3 expression by Boolean analysis. At the start of the experiment, M28z CAR T cells expressed significantly higher levels of PD-1/LAG-3/TIM-3 TP cells, and conversely, lower frequencies of PD-1/LAG-3/TIM-3 TN cells compared to MBBz CAR T cells (Sup. Figure 6b-c). The frequency of TP M28z and MBBz CAR T cells increased substantially following 24 hours of antigen exposure, especially during co-culture with MSLN^high^ OVCAR-3 cells (Figure 6c). However, in the absence of antigen stimulation (>120 hours post 1^st^ stimulation, T = 1), there was a dramatic decrease in the frequency of TP CAR T cells and conversely, the level of TN M28z and MBBz CAR T cells increased in the absence of MSLN antigen stimulation. Following re-stimulation with MSLN^high^ OVCAR-3 and SKOV-3, TP cells increased to a similar extent in both M28z and MBBz CAR T cells (Figure 6c). Of note, two distinctive CD4^+^ double positive (DP) populations were identified, PD-1/TIM3 DP cell frequencies were elevated in particularly M28z CAR T cells, while LAG-3/TIM-3 DP cells were specifically upregulated in MBBz CAR T cells. Detailed kinetics and differences in specific CIM (co-)expression patterns between M28z and MBBz CAR T cells over time during co-culture with MSLN^high^ OVCAR-3 and SKOV-3 cells can be found in Supplementary Figure 6B-D.

Substantial differences were observed in CIM co-expression in response to MSLN^high^ OVCAR-3 or SKOV-3 cells (Figure 6d). The frequency of TP (triple+) and LAG-3^+^ positive CD4^+^ and CD8^+^ CAR T cells was significantly elevated in co-cultures with MSLN^high^ OVCAR-3 compared to SKOV-3. Furthermore, the level of TIM-3^+^ CD8^+^ M28z and MBBz CAR T cells was higher following incubation with MSLN^high^ OVCAR-3 than SKOV-3. These data suggest that different target cells modulate CIM expression differentially by M28z and MBBz CAR T cells.

### Degree of trogocytosis and CIM expression levels by MSLN-CAR T cells defines specific target cell lysis-dependent clusters

**Figure 7.**
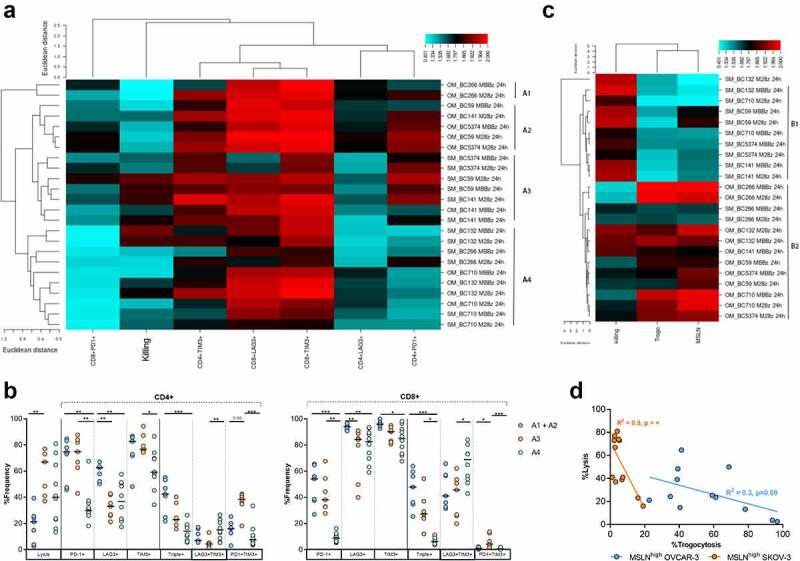
**Minimal differences detected between M28z and MBBz in CIM expression, cytolytic and trogocytotic capacity**. Unbiased hierarchical clustering of data collected through LDH assays (target cell lysis) and flow cytometry of M28z and MBBz CAR T cells, derived from six different donors, co-cultured with MSLN^high^ OVCAR-3 and SKOV-3 cells for 24 hours. n = 6 donors. A) Heatmap of unbiased hierarchial clustering of MSLN^high^ OVCAR-3 and SKOV-3 and expression of PD-1, LAG-3 and TIM-3 by M28z and MBBz CAR T cells. The Euclidian distance method and average linkage cluster algorithm were used to generate heatmap. Length of dendrogram is representative for the Euclidean distance. Columns show the %killing of target cells and CIM expression% by MSLN-CAR T cells. Rows show each individual sample as target cell_donor ID_MSLN-CAR_time. OM = MSLN^high^ OVCAR-3 and SM = MSLN^high^ SKOV-3. B) (Co-)expression of PD-1/LAG-3/TIM-3 analyzed for cluster A1+ A2, A3 and A4. C) Unbiased hierarchical clustering of MSLN^high^ OVCAR-3 and SKOV-3 target cell lysis and MSLN% on target cells and CAR T cells (Trogo). D) Linear regression analysis of %target cell lysis and %trogocytosis by M28z and MBBz CAR T cells after 24 hours of co-culture. Kruskal-Wallis tests were performed to compare between ≥3 clusters. * = P < .05, ** = P < .01, *** = P < .001. Each dot represents M28z or MBBz CAR T cells derived from 1 donor, n = 6 donors.

Target cell lysis (quantified by LDH release assays) and MSLN and CIM expression (analyzed by flow cytometry) data were generated collectively from the same experiment. In order to connect the results of these independent readouts (LDH release and flow cytometry), unbiased hierarchical clustering was performed. Unbiased hierarchical clustering of PD-1, LAG-3, and TIM-3 expression by CD4^+^ and CD8^+^ M28z and MBBz CAR T cells and lysis of MSLN^high^ OVCAR-3 and SKOV-3 cells following 24 hours of co-culture revealed four distinct clusters: A1, A2, A3, and A4 (Figure 6a). Cluster A1 (n = 2) and A2 (n = 5) solely encompassed M28z and MBBz CAR T cell donors co-cultured with MSLN^high^ OVCAR-3 cells and were characterized by low levels of OVCAR-3 lysis combined with elevated LAG-3 expression levels relative to clusters A3 and A4. Due to the large overlap between cluster A1 and A2, cluster A1 and A2 were pooled for statistical purposes in Figure 7b. Cluster A3 (n = 7) contained mainly M28z and MBBz CAR T cell donors co-cultured with MSLN^high^ SKOV-3 cells (6 out of 7) and displayed the highest level of target cell lysis. Cluster A4 (n = 10) encompassed CAR T cells incubated with either MSLN^high^ OVCAR-3 or SKOV-3 cells and was characterized by low levels of PD-1^+^ CAR T cells compared to cluster A2 and A3. The frequency of PD1^+^ CD4^+^ and CD8^+^ CAR T cells was not predictive for the degree of target cell lysis, as PD-1 expression was comparably high between low lysis cluster A1+ A2 (median lysis of 21%) and high lysis cluster A3 (median lysis of 67%), while PD-1 frequency was substantially lower in cluster A4, which displayed a wide range of lysis from 13% to 81% (median = 40%), as compared to cluster A1+ A2 and A3 (Figure 6b). As previously shown, TIM-3 expression was overall high between clusters with minimal significant differences observed, although there was a trend of lower TIM-3 expression in cluster A4 relative to cluster A1+ A2 and A3. The frequency of LAG-3/TIM-3 double positive cells was significantly lower in high target cell lysis cluster A3 compared to cluster A4 CAR T, suggesting the presence of LAG-3/TIM-3 double positive cells has a negative effect target cell lysis capacity. Conversely, PD-1/TIM-3 double positive cells were more abundant in high target cell lysis cluster A3 compared to A4 CAR T cells, indicating PD-1/TIM-3 double positive CAR T cells did not impair effective target cell lysis. Taken together, high PD-1 and TIM-3 expression levels were found on CAR T cells responsible for both low as well as high target cell lysis, suggesting the presence of these CIMs can reflect a state of poor and good T cell functionality. Whereas high CD4^+^ LAG-3^+^ CAR T cell frequency was correlated with decreased cytotoxic capacity (Sup. Figure 7a-b).

Cluster A1 was distinguishable from other clusters due to total lack of MSLN^high^ OVCAR-3 lysis; however, the CIM expression pattern was comparable with other clusters, suggesting another mechanism such as trogocytosis at play. The frequency of trogocytotic MSLN^+^ CAR T cells was exceptionally high in cluster A1 with a median of 95%, an increment of 57% compared to cluster A2 (Sup. Figure 7c). Comparison with cluster A3 and A4 is not relevant due to the previously shown lower levels of trogocytosis during co-culture with MSLN^high^ SKOV-3 cells as compared to OVCAR-3.

To further study the relation between trogocytosis and killing ability of the M28z and MBBz CAR T cells in different target cell settings, a clustering analysis including the frequency of trogocytotic MSLN^+^ CAR T cells, MSLN% within target cells and lysis of OVCAR-3 and SKOV-3 cells was performed following 24 hours of co-culture. The analysis revealed two major clusters, B1 and B2, and one individual sample (Figure 6c). Cluster B1 was composed of solely CAR T cells showing high killing ability against MSLN^high^ SKOV-3 cells (n = 9), while cluster B2 was predominantly composed of CAR T cells, with high frequency of trogocytosis, that were exposed to MSLN^high^ OVCAR-3 cells (11 out of 13). Furthermore, this clustering confirmed that the frequency of trogocytotic MSLN^+^ CAR T cells and MSLN^high^ target cells were codependent on each other. Importantly, an inverse relationship between target cell lysis and MSLN expression by both trogocytotic CAR T cells and target cells was detected. The degree of trogocytosis by MSLN-CAR T cells negatively correlated with lysis of MSLN^high^ SKOV-3 target cells, and to a lesser extent MSLN^high^ OVCAR-3 cells (Figure 6d).

Interestingly, clustering was independent of CAR construct in both cluster analyses, indicating that the differences in CIM expression and trogocytosis between M28z and MBBz CAR T cells were overruled by donor variability and target cell-dependent cytolytic response rates.

## Discussion

Currently, various early-stage MSLN-CAR T cell trials are ongoing, covering several malignancies including malignant pleural mesothelioma (MPM), pancreatic cancer, lung cancer and ovarian cancer. Safety profiles in clinical trials using MSLN-CAR T cells have been favorable, with no overt clinical evidence for on-target/off-tumor toxicities. Despite delivering promising results in preclinical models, MSLN-CAR T cell clinical response rates have thus far been marginal, with limited persistence in the majority of patients.^26–28^

Here, we demonstrate cytotoxic capacity by both CD28- and 4–1BB MSLN-CAR T cells against MSLN^+^ target cells in various *in vitro* models, albeit with different kinetics. M28z CAR T cells displayed superior MSLN^high^ SKOV-3 spheroid infiltration and lysis compared to MBBz CAR T cells. Furthermore, we underscore the importance of co-stimulation to CAR antigen sensitivity, as only the CD28-containing MSLN-CAR T cells demonstrated cytolytic capacity against parental MSLN^low^ SKOV-3 spheroids.

MSLN surface expression decreased on OVCAR-3 and SKOV-3 cells during co-culture with MSLN-CAR T cells specifically. The loss of MSLN expression was CAR-mediated and can be explained by trogocytosis of the MSLN antigen by both M28z and MBBz CAR T cells. We observed exceptionally high trogocytosis levels during co-culture of CAR T cells with MSLN^high^ OVCAR-3 cells and to a lesser extent when co-cultured with MSLN^high^ SKOV-3. Trogocytosis not only impacts antigen availability but may also decrease CAR T cell functionality due to fratricide killing and T cell exhaustion.*^14^* We demonstrated that M28z and MBBz CAR T cells were capable of fratricide killing following separation from MSLN^high^ OVCAR-3 cells, during co-culture with MSLN^high^ OVCAR-3 cells and in an experimental *in vitro* setting using MSLN^low^ and MSLN^high^ transduced T cells. Despite initial fratricide killing, functionality of trogocytotic CAR T cells recovered in the absence of the MSLN-antigen. However, these data do not exclude the possibility of trogocytosis- and fratricide killing-induced CAR T cell impairment during chronic antigen stimulation *in vivo*. In line with previous reports, we observed increased frequencies of PD-1/LAG-3/TIM-3 TP cells within trogocytotic M28z and MBBz CAR T cells, suggesting trogocytosis and T cell exhaustion can occur concomitantly.*^14^*

The degree of trogocytosis was correlated with antigen availability, suggesting high-density targets induce an increased risk for CAR T cell dysfunction due to trogocytosis. In line with this, we detected low levels of trogocytotic CAR T cells during co-cultures with parental OVCAR-3 and SKOV-3 cells, indicating that M28z and MBBz CAR T cell-mediated trogocytosis can occur in a clinical setting as MSLN expression varies among and within ovarian cancer patients. The level of trogocytosis by M28z and MBBz negatively correlated with lysis of MSLN^high^ OVCAR-3 and especially SKOV-3 cells, indicating non-lytic synapse formation between CAR T cells and target cells. Taken together, we speculate that the limited lysis of MSLN^high^ OVCAR-3 cells can partially be explained by the high levels of MSLN surface antigen followed by excessive CAR-mediated trogocytosis which in turns diverts the CAR T cells to fratricide killing. Both naïve and activated T cells are capable of trogocytosis, although activated T cells have been shown to harbor superior trogocytotic capacity,*^29,^^30^* and trogocytosis has been described as ¨an inherent feature¨ of CD4^+^ T cell activation.*^31^* The inherent trogocytotic capacity of CD4^+^ T cells may explain the elevated levels of trogocytosis we observed for CD4^+^ MSLN-CAR T cells relative to CD8^+^ MSLN-CAR T cells and are in line with previous results.*^22^* Through trogocytosis, T cells acquire antigen-presenting capacity, and trogocytosis^+^ CD4^+^ T cells have been shown to activate as well as suppress the immune function of responding cells through antigen presentation where trogocytosis^+^ regulatory T cells (Tregs) gain immunosuppressive capacity while trogocytosis^+^ T helper (Th) cells have the potential to augment immune response.*^29,^^31^* However, the immune modulatory effects trogocytosis has on trogocytosis^+^ T cells is not well known. In addition to fratricide killing, the acquisition of MSLN by CAR T cells and especially CD4^+^ CAR T cells may impact the immune response due to changes in intracellular signaling, creating either an immunosuppressive or immunostimulatory environment.

CAR-mediated bystander killing may overcome tumor antigen heterogeneity in solid tumors, however, we did not detect CAR-mediated bystander killing by either M28z or MBBz CAR T cells in our experiments. Several mechanisms have been proposed to facilitate bystander killing, including death-receptor signaling, TNF- and IFNy-mediated lysis, and epitope spreading, and our *in vitro* data do not exclude the potential of MSLN-CAR T cell-induced bystander killing in an immunostimulatory *in vivo* environment.^17,18,32^ Importantly, CAR-mediated bystander killing has previously been reported using *in vitro* and *in vivo* solid tumor models. Albeda and colleagues recently demonstrated cyclophosphamide-induced bystander killing, while spontaneous bystander killing was not detected, in an *in vivo* model using anti-mouse MSLN-CAR T cells.^17^ Furthermore, birinipant was shown to enhance HER2-CAR T cell mediated bystander killing potential *in vitro* and *in vivo* in a TNF-dependent manner.*^18^*

The lack of CAR-mediated bystander killing does not explain the lysis of parental ovarian cancer cells with low levels of MSLN surface antigen; however, previous work has demonstrated that the limited detection of cell surface antigen by flow cytometry does not necessarily imply absolute absence of cell surface antigen nor excludes recognition of low levels of surface antigen by CAR T cells.^33^ Moreover, dynamic cell surface expression of MSLN by ovarian cancer cells, as well as presence of MSLN on a protein level in OVCAR-3 and SKOV-3 cells, has previously been reported.^22,34,35^ Together these data suggest that targeting of parental OVCAR-3 and SKOV-3 cells by MSLN-CAR T cells is due to basal MSLN expression (which is largely undetectable by flow cytometry).

Our data show that the introduction of a CD28-containing MSLN-CAR construct specifically promotes an effector T cell phenotype, while transduction with 4–1BB-containing MSLN-CARs promoted the outgrowth of central memory T cells. The superior cytotoxic activity of M28z CAR T cells following antigen exposure, is in concordance with the elevated effector functions associated with the TemRA phenotype. Previous reports have demonstrated skewness toward the effector phenotype in CD28-containing CAR T cells and central memory phenotype in 4–1BB containing CAR T cells^19,36,37^ although this finding has not been universal.^38^

Unbiased hierarchical clustering linked LAG-3 expression and presence of LAG-3/TIM-3 DP cells with reduced CAR-mediated target cell lysis, while PD-1 upregulation and the PD-1/TIM-3 DP signature was associated with both low and high CAR-mediated target cell lysis. Similarly, Matsuzaki *et al* demonstrated elevated impairment of single positive LAG-3+ and double positive PD-1/LAG-3 ovarian cancer derived tumor-infiltrating and tumor-associated lymphocytes compared to single positive PD-1 lymphocytes.^39^ Furthermore, in an orthotopic in vivo model of ovarian cancer the presence of single PD-1^+^ and PD-1/TIM-3 DP cells was associated with improved survival, while frequency of PD-1/LAG-3/TIM-3 TP and LAG-3/TIM-3 DP correlated with poor survival.^22^ Taken together, these data suggest that despite earlier onset of an exhaustive phenotype in M28z CAR T cells, MBBz CAR T cells upregulated an unfavorable phenotype characterized by LAG-3/TIM-3 DP cells in response to antigen exposure. This goes hand in hand with enhanced lysis of SKOV-3 spheroids by M28z CAR T cells relative to MBBz CAR T cells, indicating that M28z CAR T cells were not functionally exhausted. Importantly, in line with previous data, we demonstrated dynamic and reversible expression of PD-1, LAG-3 and TIM-3 CIMs.^22^ Moving beyond single blockade of PD-1, our data provide a rationale for combinatorial immune checkpoint blockade of PD-1 and LAG-3 to restore MSLN-CAR T cell functionality. Combinatorial blockade of PD-1 and LAG-3 has been demonstrated to successfully augment T cell effector functions relative to single PD-1 blockade in mouse models of ovarian cancer.^40,41^ Recently, combinatorial LAG-3 and PD-1 checkpoint blockade was shown for the first time to substantially mitigate progression of metastatic melanoma compared to PD-1 blockade alone in the RELATIVITY-047 phase II/III trial.^42^ The potential beneficial effect of combinatorial TIM-3 blockade should not be excluded as triple PD-1/LAG-3/TIM-3 knockout HER2-CAR T cells displayed superior cytolytic potential compared to double PD-1/LAG-3 knockout CAR T cells in a murine model of ovarian cancer.^43^

The frequency of LAG-3^+^ MSLN-CAR T cells increased significantly during exposure to MSLN^high^ OVCAR-3 cells as compared to SKOV-3 cells. Together with the excessive trogocytosis by M28z and MBBz CAR T cells following co-culture with MSLN^high^ OVCAR-3 cells, we speculate both LAG-3 signaling and trogocytosis impede CAR-mediated target cell lysis. Recently, in an allergic asthma setting, LAG-3 was shown to mediate trogocytosis by double-negative T cells.^44^ However, SKOV-3 and OVCAR-3 cells harbor different mutations, and we can therefore not exclude other mechanisms of resistance at play.^45^

In conclusion, we demonstrate efficient lysis of MSLN^high^ target cells by both M28z and MBBz CAR T cells, although CD28-costimulated MSLN-CAR T cells demonstrated superior sensitivity against low antigen frequencies. Furthermore, the reversible antigen-dependent upregulation of PD-1 and LAG-3 provides a rationale for combination therapy with immune checkpoint blockade to rescue the cytolytic potential of MSLN-CAR T cells. We identified trogocytosis-mediated antigen loss and subsequent fratricide killing as an important mechanism further complicating successful MSLN-CAR T cell therapy. Sequential dual antigen targeting with CAR T cells as well as bystander-inducing combination therapies could aid in overcoming (trogocytosis-mediated) tumor antigen heterogeneity.^14^

## Supplementary Material

Supplemental Material

## Data Availability

All data supporting the findings of this study are presented within the article and/or supplementary materials. Additional data related to this paper may be requested from authors.
