# Supplementary material for: Trogocytosis and fratricide killing impede MSLN-directed CAR T cell functionality
Source: Oncoimmunology. 2022 Jun 28;11(1):2093426. doi: 10.1080/2162402X.2022.2093426 (PMC9313125; doi:10.1080/2162402X.2022.2093426)
Supplement: Supplemental Material [file KONI_A_2093426_SM6507.zip › Supplementary_material_figure captions.docx]

**Sup. Figure 1**. **Functional characterization of M28z and MBBz CAR T cells.** A) Transduction efficiency within CD3^+^ T cells as determined by EGFRt staining four and eight days following retroviral exposure. N=10 donors. B) Phenotype determined by CD45RA and CCR7 phenotype in CD3^+^EGFRt^+^ M28z and MBBz CAR T cells and within CD3^+^ UT cells. N=9 donors. B) IFNy, TNF, IL2 cytokine production and CD107a degranulation by CD4^+^ and CD8^+^ M28z- and MBBz- transduced T cells following 6 hours of co-culture with MSLN^high^ (M+) OVCAR-3 and K562 cells. Gating: lymphocytes 🡪 CD3+ 🡪 CD4+ or CD8+ 🡪 IL2/IFNy/TNF/CD107a. Dark grey symbols represent M28z CAR T cells, green symbols represent MBBz CAR T cells and light grey represents UT cells. Wilcoxon tests were performed to detect differences between and MBBz CAR constructs. * = P<0.05 and ** = P<0.01. Each dot represents 1 donor.

**Sup. Figure 2**. **Lysis and infiltration of of MSLN^high^ SKOV-3 spheroids by M28z and MBBz CAR T cells.** A) MSLN^high^ SKOV-3 target cell lysis as determined by LDH release following co-culture with CD4^+^ enriched, CD8^+^ enriched and unsorted CD4^+^/CD8^+^ M28z and MBBz CAR T cells at a 1:5 or 2:1 effector to target ratio for 24 hours. N=6 donors, each dot represents one donor. B) Highest caspase3/7 signal during 24 hours of co-culture with MSLN^high^ SKOV-3 spheroids and M28z or MBBz transduced T cells. GCU=green calibrated unit C) EGFRt% and D) CD4% and CD8% in M28z and MBBz transduced T cells prior to start of co-cultures with SKOV-3 spheroids.  E) Fold change in CD4^+^ and CD8^+^ T cell frequency within the SKOV-3 spheroids and supernatant as determined by flow cytometry relative to T=0. Fig. A, C-E, each dot represents one donor, n= 6 donors. Fig. B, each dot represents the mean of 3 technical replicates of 1 donor, n=6 donors. Grey symbols represent M28z CAR T cells and green symbols represent MBBz CAR T cells. To compare target cell lysis by CD4^+^ vs CD8^+^ vs CD4^+^/CD8^+^ CAR T cells, Friedman tests were performed.  Wilcoxon tests were used to compare between M28z and MBBz. * = P<0.05.

**Sup. Figure 3. Lysis of target cells with different MSLN expression by M28z and MBBz CAR T cells.** A) MSLN frequency on MSLN^high^ OVCAR-3 and SKOV-3 cells (right) and MFI of MSLN^+^ cells upon start of co-culture. Each dot represents an independent experiment. B) Representative MSLN expression on MSLN^high^ OVCAR-3 and SKOV-3 cells upon start of co-culture and following 4 hours of incubation. 1 out of 6 representative donors is shown. C) MSLN frequency within GFP^+^ target cells after 4 and 24 hours of incubation with UT, M28z and MBBz cells. N=6 donors, each dot represents one donor D) Highest caspase3/7 signal during 24 hours of co-culture with MSLN^high/low^ and MSLN^low^ SKOV-3 spheroids and M28z or MBBz transduced T cells. GCU=green calibrated unit. Each dot represents the mean of 3 technical replicates from 1 donor, n=6 donors. E) Graphical illustration of bystander killing as detected by F) Chromium51 (Cr51) release (each dot represents the mean of 3 technical replicates from 1 donor, n=5 donors) or G) CellTrace Violet (CTV) signal (each dot represents the mean of 2 technical replicates from 1 donor, n=2-3 donors). Dark grey symbols represent M28z CAR T cells, green symbols represent MBBz CAR T cells and light grey represents UT cells. Friedman tests were performed to compare between 3 different target cell conditions or T cell treatments (UT vs M28z vs MBBz). To study differences between M28z and MBBz, Wilcoxon tests were used. * = P<0.05 and ** = P<0.01.

**Sup. Figure 4**. **MSLN CAR T cells display trogocytotic and fratricide killing capacity.** A) Representative gating for quantifying MSLN expression by target cells and CAR T cells. 1 out of 6 representative donors is shown, following 4 hours of co-culture. B) Frequency of MSLN within CD3^+^CAR^+^ M28z and MBBz CAR T cells or CD3^+^CAR^˗^ UT cells following 4 hours of co-culture with MSLN^high^ OVCAR-3 and SKOV-3 cells. C) MSLN expression within CAR^+^ and CAR^˗^ M28z and MBBz transduced T cells. CAR expression was determined by hFAB staining. D) MSLN expression by M28z and MBBz CAR T cells following co-culture with parental and MSLN^high^ OVCAR-3 and SKOV-3 cells. E) MSLN expression within CD4^+^CAR^+^ and CD8^+^CAR^+^ CAR T cells during co-culture with MSLN^high^ OVCAR-3 and SKOV-3 cells. Each dot represents one donor, n =6 donors. F) MSLN expression within EGFRt^+^ trogo^+^ or trogo^˗^ sorted T cells, one and four days post sort. G) Frequency of unproliferated (passage 0) CTV^+^EGFRt^+^MSLN^+/˗^ sorted T cells, four days after sorting. Each dot represents M28z or MBBz CAR T cells from 1 donor, n=3 donors. H) The MSLN frequency on M28z and MBBz CAR T cells (EGFRt^+^) following co-culture with MSLN^high^ OVCAR-3 target cells without pre-treatment or pre-treated with vehicle (DMSO) or Latrunculin A (LatA). Each dot represents M28z or MBBz CAR T cells from one donor, n=3-4 donors. Dark grey symbols represent M28z CAR T cells, green symbols represent MBBz CAR T cells and light grey represents UT cells. Friedman tests were performed for comparison between T cells (UT vs M28z vs MBBz) or treatments (target cells only vs DMSO vs LatA). Wilcoxon tests were used to compare within one CAR construct. * = P<0.05, ** = P<0.01.

**Sup. Figure 5.** **MSLN CAR T cells display fratricide killing capacity.** A) Representative plots of MSLN frequency within MSLN-transduced and sorted T cells. 1 out of 5 donors displayed. B) The MSLN frequency within CD3^+^EGFRt^+^ M28z- and MBBz-transduced T cells was monitored during co-culture with MSLN^high^, MSLN^low^ and mixed MSLN^high/ow^ autologous T cells. Each dot represents 1 donor, n=5 donors. C) Viability of MSLN^high^ OVCAR-3 cells following overnight co-culture with CTV^+^ M28z or MBBz CAR T cells and freshly added CTV^˗^ CAR T cells (+CAR) or UT cells (+UT). D) Lysis of MSLN^high^ OVCAR-3 cells in response to addition of fresh CTV- CAR T cells as compared to UT cells. E) MSLN expression within CTV^˗^EGFRt^+^ CAR T cells. Each dot represents M28z or MBBz CAR T cells from one donor, n=4 donors. Grey symbols represent M28z CAR T cells and green symbols represent MBBz CAR T cells. Friedman tests were performed for comparison between target cells (MSLN^high^ vs MSLN^high/low^ vs MSLN^low^). Wilcoxon tests were used to compare between and within CAR constructs. * = P<0.05 and ** = P<0.01.

**Sup. Figure 6. CIM (co-)expression on MSLN CAR T cells during co-culture with MSLN^high^ target cells.** A)  Kinetics of PD-1, LAG-3 and TIM-3 frequency (top) and MFI (bottom) within CD4^+^ or CD8^+^ CAR+ M28z and MBBz transduced T cells prior (0h) and during co-culture (4h and 24h) with MSLN^high^ SKOV-3 cells. Single and co-expression profile of PD-1/LAG-3/TIM-3 CIMs overtime on B) CD4^+^ and C) CD8^+^ M28z and MBBz CAR T cells. Each dot represents one donor, n=6 donors. D) Kinetics of triple negative (TN) T cell frequency within CAR^+^ M28z and MBBz transduced cells during the experiment. Median of 6 donors is displayed. Grey symbols represent M28z CAR T cells and green symbols represent MBBz CAR T cells. Wilcoxon tests were performed to compare between two CAR constructs. Friedman tests were used per CAR construct over time (≥3 timepoints). * = P<0.05, ** = P<0.01.

**Sup. figure 7. Link between target cell lysis and LAG-3 expression and trogocytosis by MSLN-CAR T cells.** A) Linear regression analysis between MSLN^high^ cell lysis (OVCAR-3 + SKOV-3) and LAG-3 expression by CD4^+^ or CD8^+^ CAR T cells. B) Linear regression analysis between MSLN^high^OVCAR-3 or SKOV-3 target cell lysis and LAG-3 expression by CD4^+^ CAR T cells. C) MSLN expression by CAR T cells (trogocytosis+) after 24 hours of exposure to MSLN^high^ target cells, analyzed for cluster A1, A2, A3 and A4. Kruskal-Wallis tests were performed to compare between ≥3 clusters. * = P<0.05, ** = P<0.01, *** = P<0.001. Each dot represents M28z or MBBz CAR T cells derived from 1 donor, n=6 donors.
