# Supplementary material for: Trogocytosis and fratricide killing impede MSLN-directed CAR T cell functionality
Source: Oncoimmunology. 2022 Jun 28;11(1):2093426. doi: 10.1080/2162402X.2022.2093426 (PMC9313125; doi:10.1080/2162402X.2022.2093426)
Supplement: Supplemental Material [file KONI_A_2093426_SM6507.zip › Supplementary_material_figure1_220616.docx]

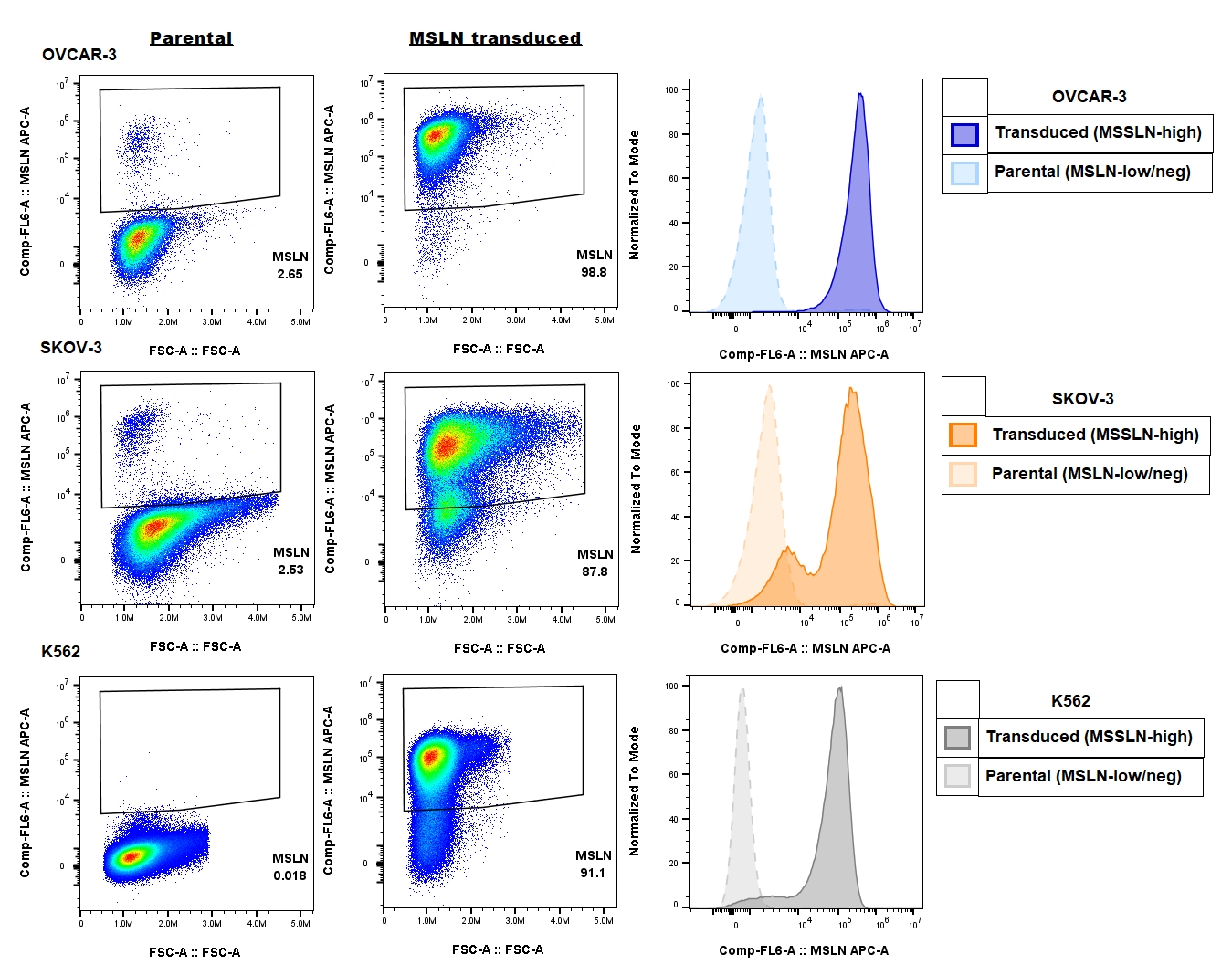


**B**

**A**

**Supplementary Material 1**


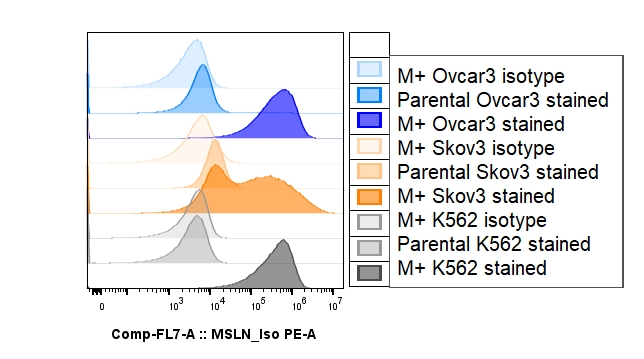
**Supplementary Material 1**. **Transduction of parental target cells with MSLN.** A) Representative FACS plots of MSLN frequency in parental (MSLN^low/neg^) and MSLN-transduced and sorted (MSLN^high^) OVCAR-3, SKOV-3 and K562 cells. 1 out of 6 individual experiments is shown. B) Staggered overlay of parental and MSLN^high^ (M+) cell lines stained with MSLN-PE (FAB32652P) or corresponding isotype control.
