# Supplementary figures and images for: Trogocytosis and fratricide killing impede MSLN-directed CAR T cell functionality
Source: Oncoimmunology. 2022 Jun 28;11(1):2093426. doi: 10.1080/2162402X.2022.2093426 (PMC9313125; doi:10.1080/2162402X.2022.2093426)

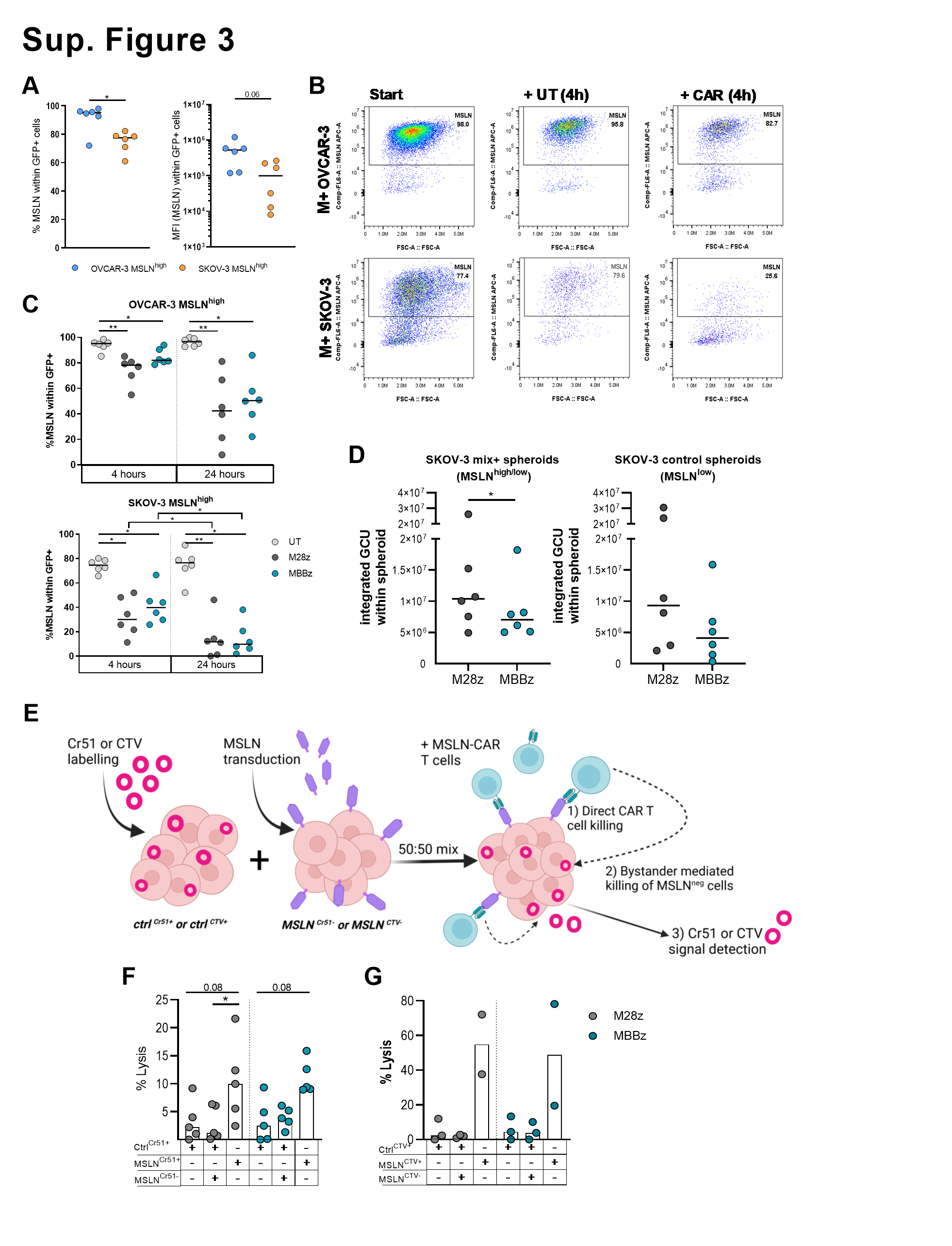

Supplement: Supplemental Material [file KONI_A_2093426_SM6507.zip › Sup. Fig 3.tif]

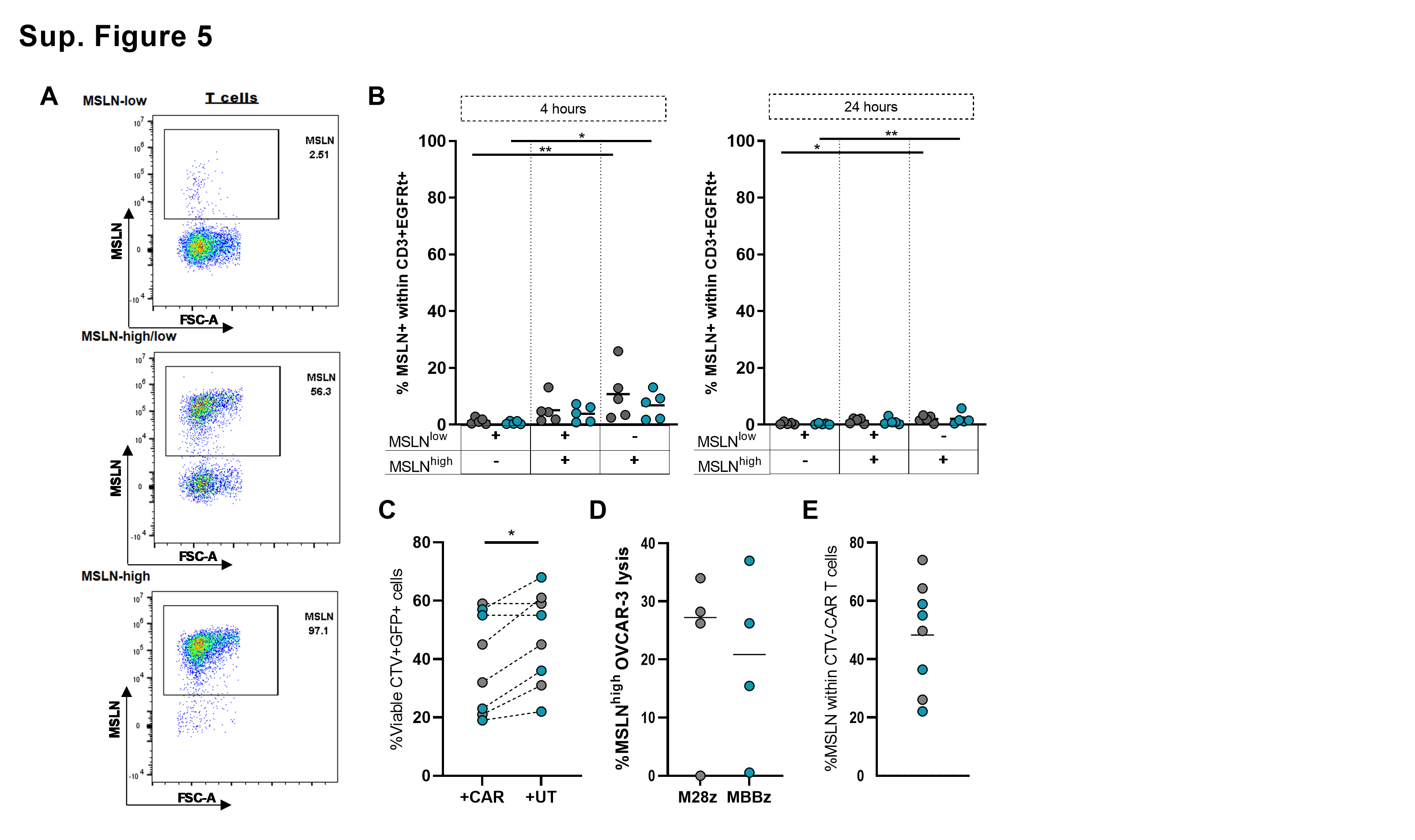

Supplement: Supplemental Material [file KONI_A_2093426_SM6507.zip › Sup. Fig. 5.tif]

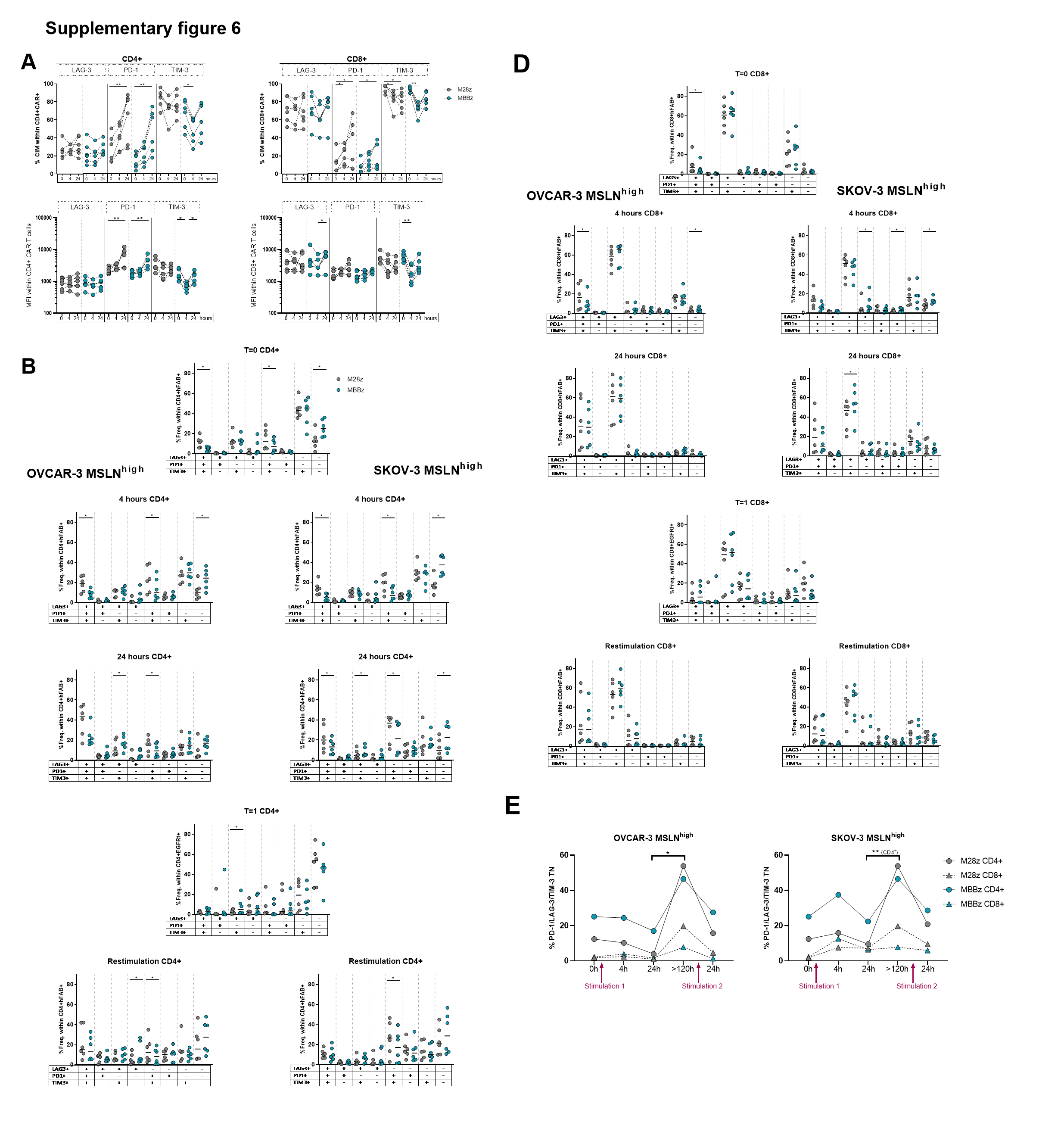

Supplement: Supplemental Material [file KONI_A_2093426_SM6507.zip › Sup. Fig. 6.tif]

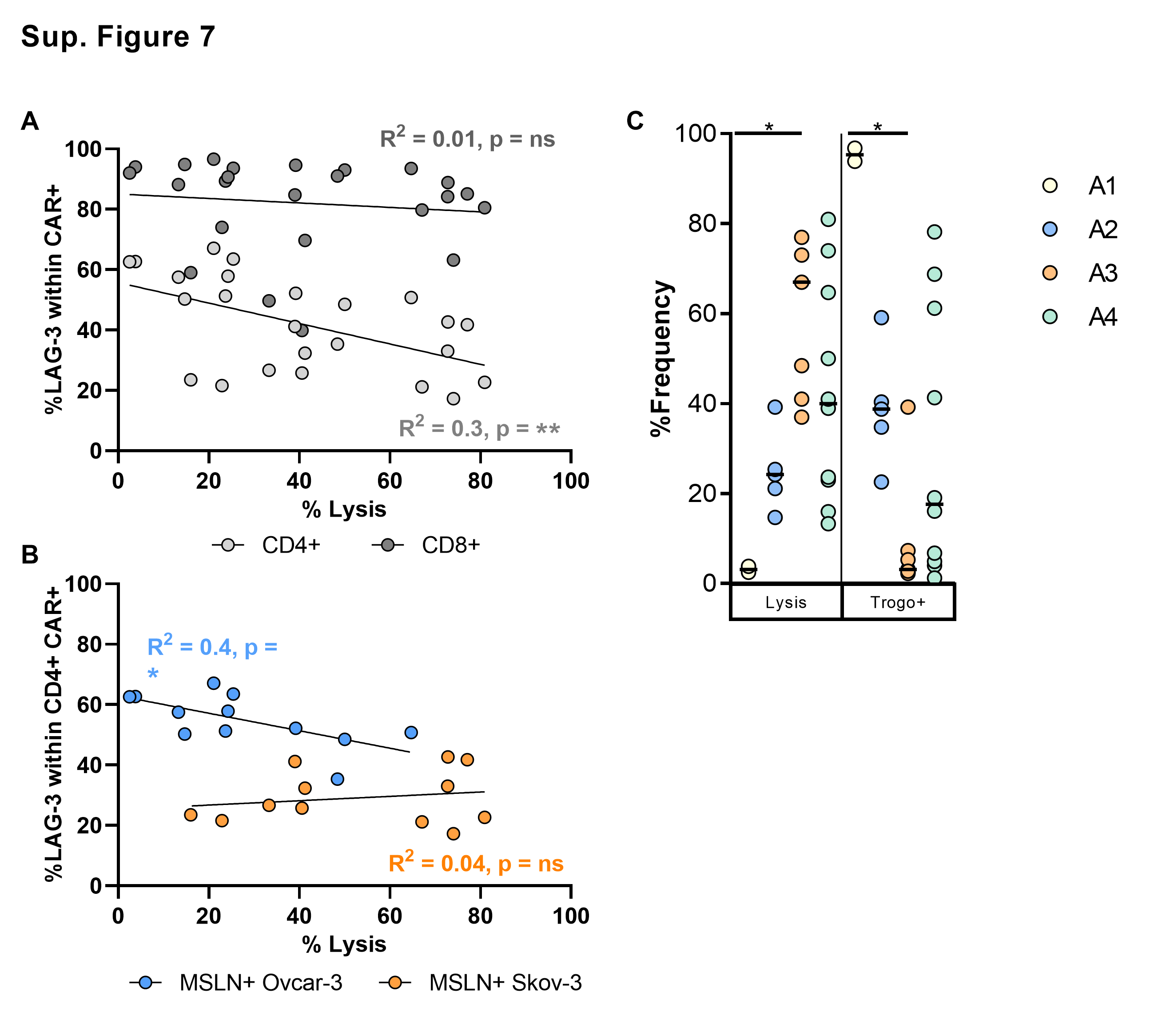

Supplement: Supplemental Material [file KONI_A_2093426_SM6507.zip › Sup. Fig. 7.tif]

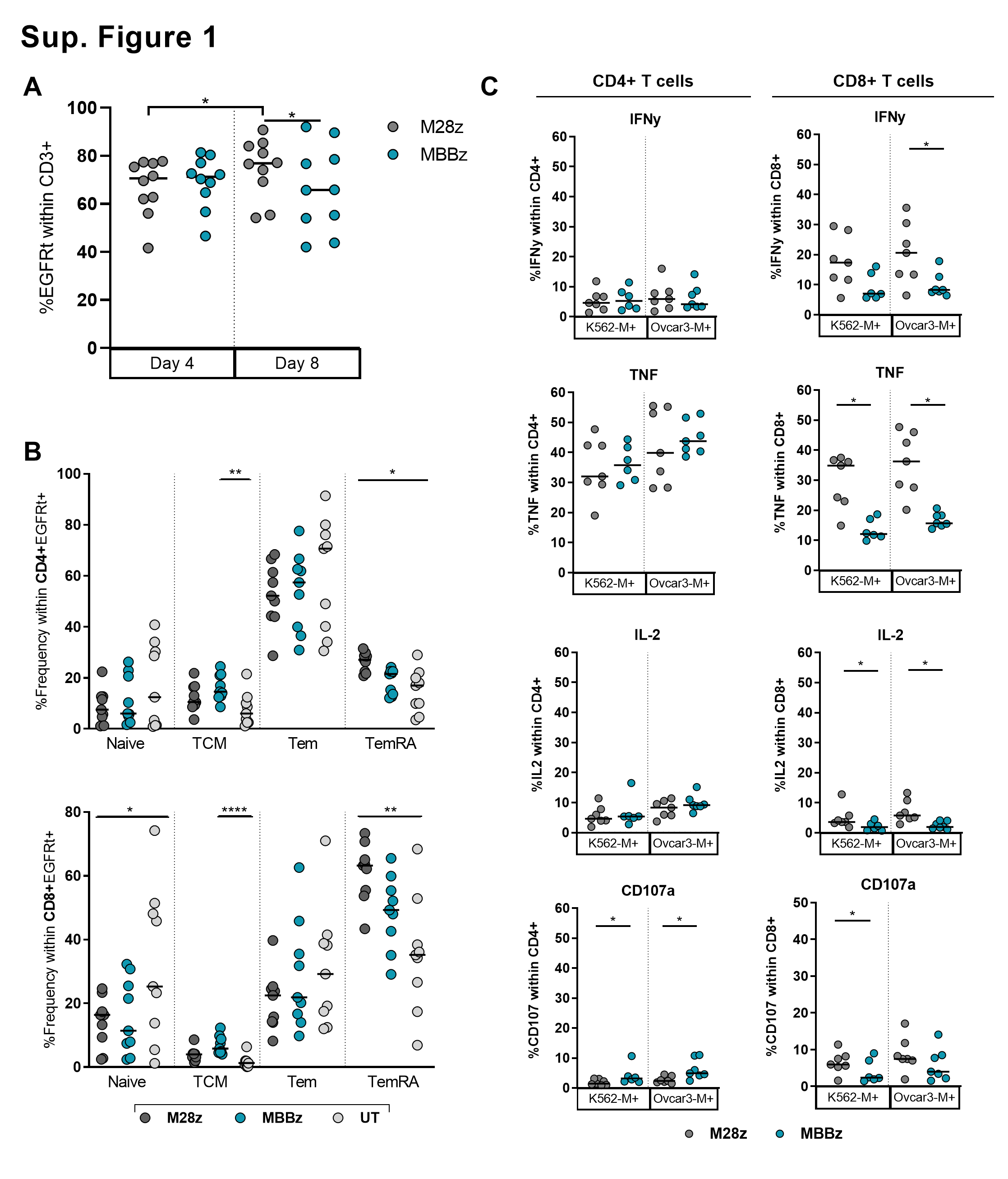

Supplement: Supplemental Material [file KONI_A_2093426_SM6507.zip › Sup. Figure 1.tif]

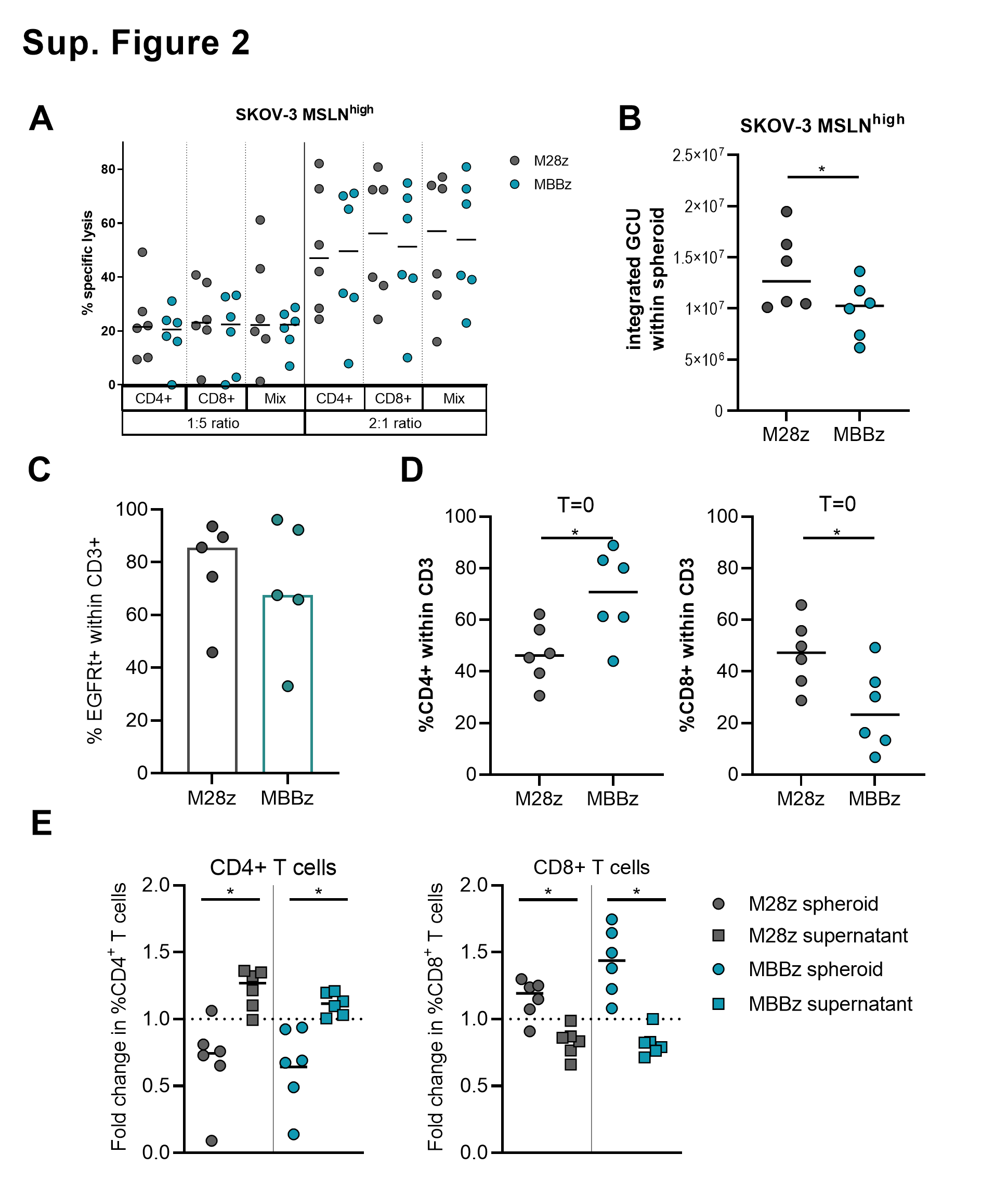

Supplement: Supplemental Material [file KONI_A_2093426_SM6507.zip › Sup. Figure 2.tif]

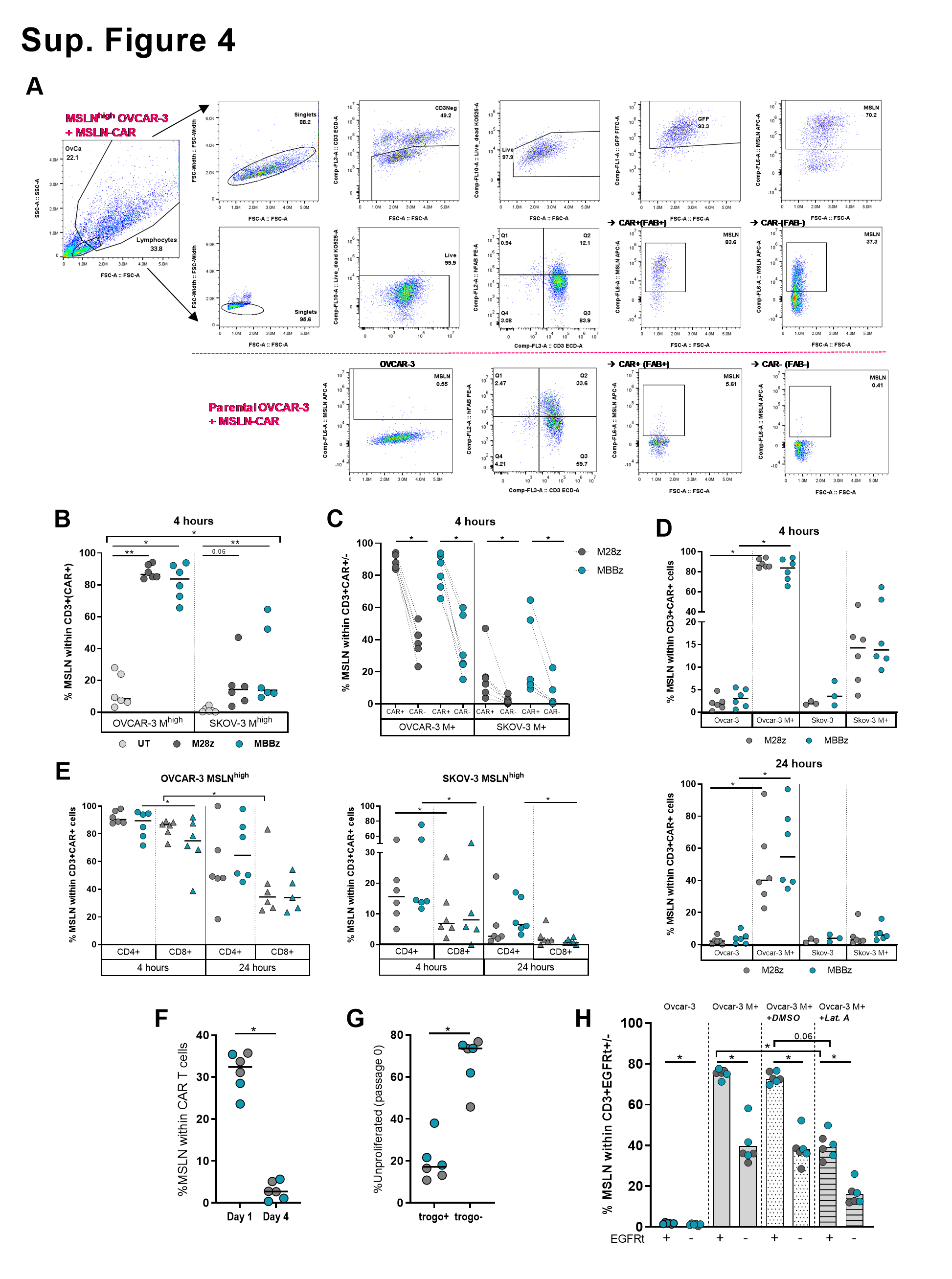

Supplement: Supplemental Material [file KONI_A_2093426_SM6507.zip › Sup. Figure 4.tif]
